# Molecular quantification of fritillariae cirrhosae bulbus and its adulterants

**DOI:** 10.1186/s13020-024-01010-z

**Published:** 2024-10-08

**Authors:** Ziyi Liu, Yifei Pei, Tiezhu Chen, Zemin Yang, Wenjun Jiang, Xue Feng, Xiwen Li

**Affiliations:** 1 State Key Laboratory for Quality Ensurance and Sustainable Use of Dao-Di Herbs, Institute of Chinese Materia Medica, China Academy of Chinese Medical Sciences, Beijing, 100700 China; 2 Sichuan Provincial Key Laboratory of Quality and Innovation Research of Chinese Materia Medica, Sichuan Academy of Chinese Medicine Sciences, Chengdu, 610041 China

**Keywords:** Pyrosequencing, Adulteration, Chinese patent medicines, Molecular quantitative, Single nucleotide polymorphisms

## Abstract

**Background:**

Fritillariae Cirrhosae Bulbus (FCB) is frequently adulterated with its closely related species due to personal or non-man made factors, leading to alterations in the composition of its constituents and compromising the efficacy of its products.

**Methods:**

The specific single nucleotide polymorphisms (SNPs) were screened by comparing candidate barcodes of *Fritillaria* and verified by amplification and sequencing. Herb molecular quantification (Herb-Q) was established by detecting specific SNPs, and the methodological validation was performed. Quantitative standard curves were established for FCB mixed with each adulterated species, and the quantitative validity of this method was verified based on external standard substance. In addition, eight commercial Shedan Chuanbei capsules (SDCBs) randomly selected were detected.

**Results:**

FCB and its five adulterants can be distinguished based on the ITS 341 site. The methodological investigation of Herb-Q shows optimal accuracy, and repeatability, which exhibited good linearity with an R^2^ of 0.9997 (> 0.99). An average bias in quantitative validity was 5.973% between the measured and actual values. Four of eight commercial SDCBs were adulterated with *F. ussuriensis* or *F. thunbergia* with adulteration levels ranging from 9 to 15% of the total weight.

**Conclusion:**

This study confirmed that Herb-Q can quantitatively detect both the mixed herbs and Chinese patent medicines (CPMs) containing FCB with high reproducibility and accuracy. This method provides technical support for market regulation and helps safeguard patient rights.

**Supplementary Information:**

The online version contains supplementary material available at 10.1186/s13020-024-01010-z.

## Introduction

Up to 80% of the population in Asian and African countries relies on herbal medicines (HMs) for primary health care [1]. In addition, HMs have shown great advantages in the treatment of many persistent diseases [2–4]. However, adulteration in the trade of HMs has become widespread owing to various regulatory challenges and objective factors, jeopardizing their quality and purity [5]. Purity is one of the requirements of the World Health Organization (WHO) guidelines, which require that foreign matter (including other species of plants) in a sample should not exceed limits set in national, regional, or international pharmacopoeias [6, 7]. The Chinese Pharmacopoeia requires that impurities (including adulterants) in herbs and decoction pieces should not exceed 3%. Accurately determining the weight of target species in HMs to ensure that the amounts of raw materials meet the requirements can guarantee their clinical efficacy, which is also one of the important tasks of drug supervision departments. Therefore, a rapid, accurate, and easy-to-promote species identification and quantitative detection assay will greatly help promote the quality control of the raw materials at the species level of HMs products and provide an effective way for market supervision and international trade. In China, the multi-source traditional Chinese medicine Fritillariae Cirrhosae Bulbus (FCB), which has been approved to come from six species of the genus *Fritillaria*, is an antitussive and expectorant HM widely used in Chinese patent medicines (CPMs), modern herbal formulas, and food treatments, with significant therapeutic effects in treating cough and asthma, eliminating phlegm, and resisting tumor effects [8–10]. Owing to differences in efficacy, supply, and demand, the price of FCB is 10–200 times higher than that of other *Fritillaria* bulbs [11]. *Fritillaria* species exhibit a high degree of similarity in their apparent morphology of medicinal parts. Traders sometimes mix it with cheaper closely related species to make a profit in the market, and the common adulterants include *F. hupehensis* Hsiao et K. C. Hsia, *F. thunbergii* Miq., *F. ussuriensis* Maxim., and Fritillariae pallidiflorae bulbus (FPB) [12, 13]. The dosage of different *Fritillariae* species in CPMs varies depending on their chemical properties and pharmacological effects [14–16]. Adulteration of FCB not only affects legal trade but also influences the effectiveness of CPMs. Therefore, the amount of FCB and its adulterants urgently required to be detected to ensure authenticity, clinical safety, and traceability.

The identification of FCB includes species authentication and quantitative testing. DNA barcoding is an effective species identification method for FCB, but it cannot be used for quantifying [17, 18]. Currently, physical, chemical, and molecular identification methods are mainly used to quantitatively identify adulteration by FCB [12, 19–21]. Near infrared spectroscopy (NIR) combined with linear discriminant analysis (LDA) and partial least squares (PLS) techniques can detect the mixing ratio of FCB and forgeries, with a minimum of 5% forgeries [19]. Mixed samples of *F. cirrhosa* and its adulterants were analyzed using laser-induced breakdown spectroscopy (LIBS), and it was found that the adulterants accounted for at least 5% of the samples [12]. However, these methods require the learning effect of the model to be highly correlated with the input dataset, which is highly dependent on sample size and source, limiting the application of them to different samples [22]. Quantitative techniques based on PCR methods have been commonly used for the identification of target herbs in HM products, such as the identification of *Panax notoginseng* powder and *Ligusticum chuanxiong* in ChuanXiong ChaTiao Wan [23, 24]. However, these methods are suitable for species with large differences in gene sequences and have certain limitations in quantifying closely related species [25]. Herb molecular quantification (Herb-Q) method is one of the few technologies that can quantify single nucleotide polymorphisms (SNPs) in species from the same genus [26]. It has preliminary quantified *Pinellia ternata* and its low-taxonomic-level adulterants, confirming the potential to quantify HMs. We propose to quantitatively identify FCB and its adulterants by Herb-Q and attempt to apply it to the detection of CPMs.

Herein, an herb molecular quantification (Herb-Q) method was applied to quantify FCB and its common counterfeits. Specific SNPs for distinguishing FCB and its five adulterants were screened and identified based on commonly used candidate DNA barcodes. The weight of FCB and each adulterant can be calculated by detecting the fluorescence signal value of the target sites. Herb-Q was comprehensively tested, and its quantitative ability was assessed by measuring the bias with external standard substances. We also evaluated the effectiveness of Herb-Q as a quantitative method for HMs using self-produced Shedan Chuanbei capsule (SDCB), and applied into the quantitative determination of commercial SDCBs. This study not only identified SNPs for quantitative detection of FCB and its common adulterants, verified the applicability and accuracy of the Herb-Q assay for the identification and quantitative detection of adulteration of FCB at the species level, but also verified the applicability of this method in patent medicine products for the first time.

## Materials and methods

### Sample collection

Chinese Pharmacopoeia contains six original species of FCB: *Fritillaria cirrhosa* D. Don, *F. unibracteata* Hsiao et K. C. Hsia, *F. przewalskii* Maxim., *F. delavayi* Franch., *F. taipaiensis* P. Y. Li, and *F. unibracteata* Hsiao et K. C. Hsia *var. wabuensis* (S. Y.Tang et S. C. Yue)Z. D. Liu, S.Wanget S. C. Chen. It also contains five adulterants: *F. hupehensis*, *F. ussuriensis*, *F. thunbergii*, and FPB (*F. pallidiflora* Schrenk and *F. walujewii* Regel). A total of 170 plants were collected from ten provinces in China (Supplementary Table 1). To confirm the authenticity of the collected samples, the ITS sequences obtained by Sanger sequencing were analyzed using BLAST at the National Center for Biotechnology Information (NCBI). All the samples collected in this study have been appraised by Professor Xiwen Li from the Institute of Chinese Materia Medica, China Academy of Chinese Medicinal Sciences, Beijing, China. Commercially available CPMs included eight SDCBs purchased from three pharmacies in Guangdong, Tianjin, and Beijing, and the samples were named 1–8, respectively. The ingredients in the self-produced SDCB were verified samples, which were prepared according to the instructions of Chinese Pharmacopoeia (2020 edition, Part I).

### DNA extraction, PCR amplification, and sanger sequencing

The collected samples were dried at 50 ℃ for 12 h to remove excess water, and the dried bulbs were ground into powder by mortar. Briefly, 200 mg of each powder were weighed and used as voucher samples. Genomic DNA was extracted using a Hi-DNA Secure Plant Kit (Tiangen, China) following the manufacturer’s protocol. Concentration and purity of the extracted DNA were detected by NanoDrop 2000 (Thermo Fisher Scientific, China) and stored at 4 ℃. DNA templates from voucher samples were used to amplify ITS and *mat*K sequences using universal primers (ITS S2F/S3R and *mat*K 390F/1326R) [27]. The PCR products were sequenced by Sangon Biotech Co., Ltd. (Shanghai, China).

### SNP screening and verification

The ITS, *psb*A-*trn*H, *mat*K, and *rbc*L sequences of *Fritillaria* were downloaded from the GenBank database of NCBI (https://www.ncbi.nlm.nih.gov/) for preliminary screening of sequence conservation and applicability. Suitable sequences were screened, combined with self-test sequences, and aligned using the CodonCode software (CodonCode Co., USA). After proofreading, candidate SNPs of the 11 species of *Fritillaria* were analyzed. According to the sequencing procedure of the PyroMark^®^ Q48 instrument [28], the reactants are composed of PCR products and magnetic beads. DNTPs, sequencing primers, enzymes, substrates, and buffers were added to the reactants using specific reaction procedures for complete sequencing. To prepare high-quality PCR products for Herb-Q, specific SNPs, amplification forward primers, reverse primers, and pyrosequencing primers were screened and designed according to the PyroMark Q48 guidelines. A pair of specific PCR and sequencing primers was designed according to SNPs (Supplementary Table 2). The primers were synthesized by Sangon Biotech Co., Ltd. (Shanghai, China).

### Developmental validation of Herb-Q

The linearity, limit of detection (LOD), limit of quantification (LOQ), repeatability, and accuracy of Herb-Q were validated using a randomly selected specific SNP for verification sites. To complete Herb-Q validation, mixed powders were prepared from two randomly selected species, including one original species and one adulterant of FCB.

#### Linearity, LOD and LOQ determination

FCB and *F. hupehensis* were randomly selected as genuine and counterfeit products, respectively. Mixed powders were prepared by adding 1, 2, 4, 6, 8, 10, 20, 30, 40, and 50% (wt/wt) counterfeit products to genuine products. Three technical replicates were performed for each sample and these ten proportions were selected to establish a linear regression equation. The linear relationship between species proportion and allelic frequency was explored, and the dynamic range was determined. The LOD and LOQ of Herb-Q were evaluated by mixing counterfeit powder with genuine powder at concentrations of 1, 2, 4, 6, and 8%. The LOD was defined as the stable detection ≥ 95%, while LOQ determination was confirmed based on the relative standard deviation (RSD) ≤ 25%; 20 replicates were performed for each DNA extraction of mixed powder samples with different adulteration ratios [23].

#### Repeatability and accuracy

To verify the accuracy and applicability of Herb-Q, mixed powders of FCB with detection ratios of 65, 75, and 85%, and *F. hupehensis* with corresponding proportions of 35, 25, and 15% were used, and six technical replicates were performed for each sample [29].

### Quantitative detection by Herb-Q

A good linear relationship was key to successfully constructing Herb-Q. In this study, an Herb-Q system was established by constructing a good quantitative standard linear relationship combined with external standard substances.

#### Establishment of the quantitative standard curve

Mixed samples of FCB and *F. ussuriensis*, which was selected as counterfeit product, were prepared at ratios of 0:100, 10:90, 30:70, 50:50, 70:30, 90:10, and 100%:0% (wt/wt), and a quantitative standard curve for specific SNP was established. Three technical replicates were performed for each sample. The curve was considered to have a good linear relationship when the determining coefficient (R^2^) ≥ 0.99 [30].

#### Quantitative calculation

To validate the feasibility and accuracy of Herb-Q, external standard substances with known weights were used for the mixed samples. FCB, *F. ussuriensis*, non-adulterated impurities, and a known amount of *F. thunbergii* were mixed into three portions of approximately 200 mg, in which the non-adulterated impurities were the three cases containing soil, wheat-flour, or both soil and wheat-flour. Using this sample as an example, the actual total amount of FCB was measured as the target species. The first step was to calculate the actual total weight by dividing the known weight of *F. thunbergii* by the corresponding allele frequency, as shown in Eq. (1). The second step was to calculate the actual weight of FCB by multiplying the actual total weight with the allele frequency of FCB, as shown in Eq. (2) [26].

1 $$W\_FCB = W\_thunbergii / R\_thunbergii$$

2 $$W\_tar = W\_FCB \times \, R\_tar$$

where W_thunbergii represents the actual weight of *F. thunbergii* in the test sample; R_thunbergii is the ratio of the chemiluminescent signal of a unique genotype for *F. thunbergii* at a specific SNP locus; W_FCB is the calculated total amounts of FCB; and R_tar is the ratio of the chemiluminescent signal of a unique genotype for target species at the specific SNP locus.”

Herb-Q was considered to be successfully constructed when the bias between the calculated and actual values were less than 25% [24].

### Quantitative detection of CPMs by Herb-Q

To ensure the accuracy of the established quantitative method for detecting CPMs, FCB, *F. ussuriensis*, and snake bile were mixed to prepare self-produced SDCB. Approximately 0.1 g of the self-produced CPM was weighed, and an equal proportion of *F. thunbergii* was added as an external standard substance to form a mixed powder. The quantitative construction steps calculated the actual weights of FCB and adulterants (*F. ussuriensis*). The quantitative method for CPMs was considered successful when the bias between the calculated and actual values was less than 25% [24]. Eight batches of commercial SDCBs were quantitatively analyzed. First, samples were collected separately for DNA extraction, and specific SNPs were used to confirm the composition of each sample to determine the type of external standard substances to be added. The samples and external standard substances were then combined to form mixed powders. The mixed powders were identified according to the quantitative steps, and the weights of FCB and adulterants in each batch of samples were quantified.

## Results

### Sequence selection and collection

Sequence conservation and SNP specificity are the basis of Herb-Q assay [31]. To select candidate SNPs, suitable sequences were identified using commonly used candidate barcodes (ITS, *mat*K, *rbc*L, and *psb*A-*trn*H). A total of 734 ITS, 232 *psb*A-*trn*H, 421 *mat*K, and 382 *rbc*L sequences from *Fritillaria* were downloaded from GenBank database. The phylogenetic tree was established to complete the phylogenetic analysis after aligning the sequences, and sequences with incomplete sequencing or obviously abnormal evolutionary positions were eliminated. A total of 467 ITS, 168 *psb*A-*trn*H, 320 *mat*K, and 284 *rbc*L sequences were suitable for subsequent SNPs screening. Based on the specificity of SNPs, no suitable SNPs were screened in the *rbc*L and *psb*A-*trn*H sequences. Hence, the 467 ITS and 320 *mat*K sequences were determined suitable for quantifying FCB and its adulterants using Herb-Q. The ITS and *mat*K sequences from 170 samples were examined and collected as self-test sequences to complement the reference database used for Herb-Q analysis. Combined with previously downloaded sequences, a total of 637 ITS and 490 *mat*K sequences were aligned to screen for candidate SNPs (Supplementary Table 3).

### Screening of candidate SNPs

**Fig. 1:**
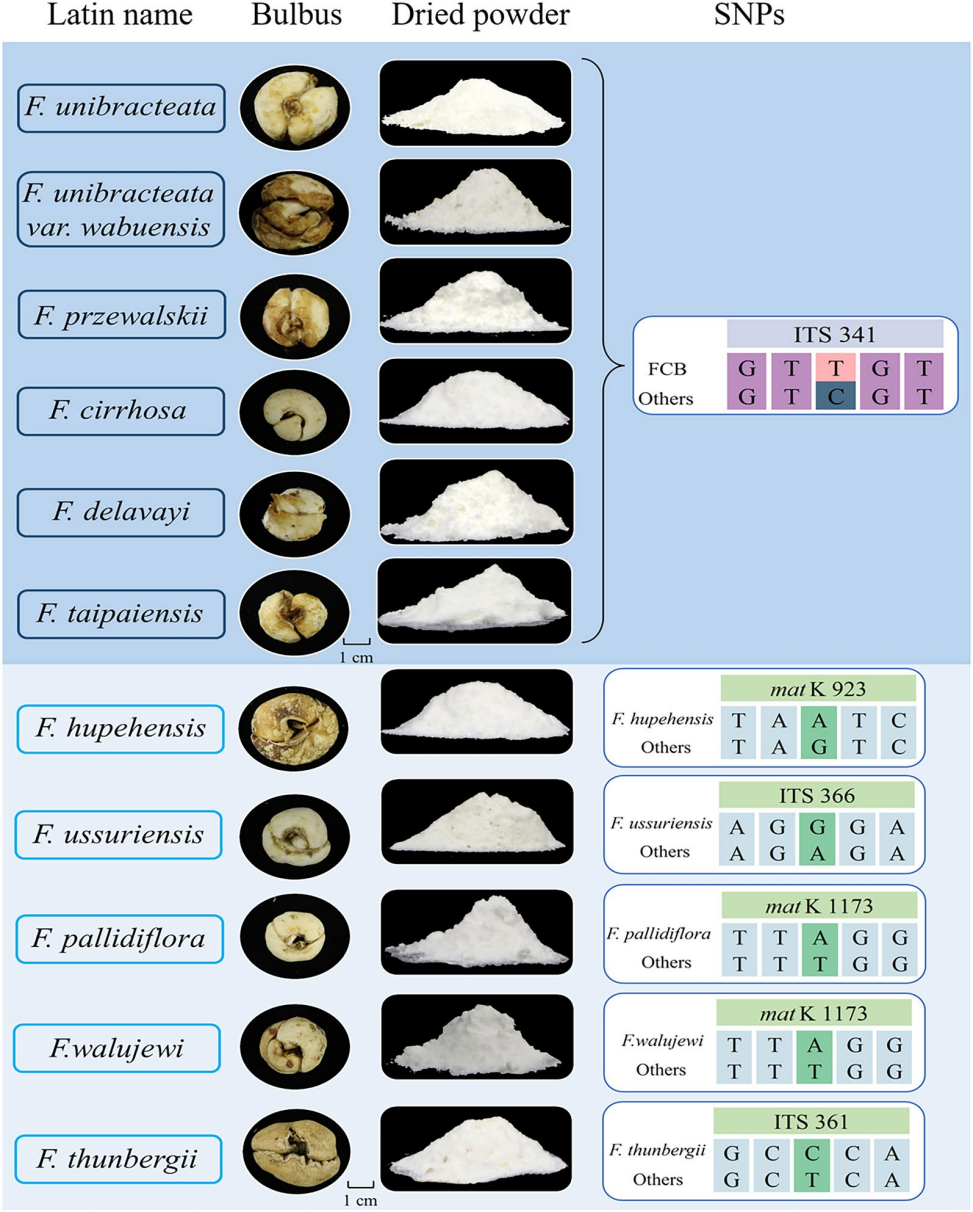
Characteristics and SNPs information of Fritillariae Cirrhosae Bulbus (FCB) and its adulterants. From left to right, the Latin names, medicinal parts, dried powder and SNP of six original species and five adulterants of FCB are shown

Five SNPs that could identify FCB and its five adulterants were obtained following the two-step screening criteria. First, to screen the identification sites of FCB and its adulterants, the sequences of six original species of FCB were aligned with the sequences of five adulterated species, and ITS 341 and *mat*K 336 site were obtained. At ITS 341 site, all species were genotyped as T or C, while at *mat*K 336 site, all species were genotyped as A or G (Supplementary Table 4). Based on the principles of pyrosequencing instrument, base A consumes energy during the experimental reaction, which affects the quantitative results. Therefore, ITS 341 site was more suitable for further validation and was chosen as the identification site for FCB and its adulterants (Fig. 1). Second, to screen the identification sites of five adulterants of FCB, the sequences of single adulterant species were compared with those of other *Fritillaria* species (Supplementary Table 5). The ITS 361, ITS 366, *mat*K 923, and *mat*K 1173 sites could be used to identify *F. thunbergii*, *F. ussuriensis*, *F. hupehensis*, and FPB, respectively, which corresponded to genotypes C, G, A, and A (Fig. 1). The screened sites were unique identification sites; therefore, the energy consumption of the bases was not considered. Five candidate SNPs were obtained by preliminary screening, and their specificity and effectiveness require further verification.

### Verification of specificity and validity of candidate SNPs

To verify the specificity of the five SNPs obtained from the screening, a 55 bp sequence containing the SNP was selected [32]. BLAST results for these sequence sites were consistent with those of the corresponding identified species (Supplementary Table 6).

**Fig. 2:**
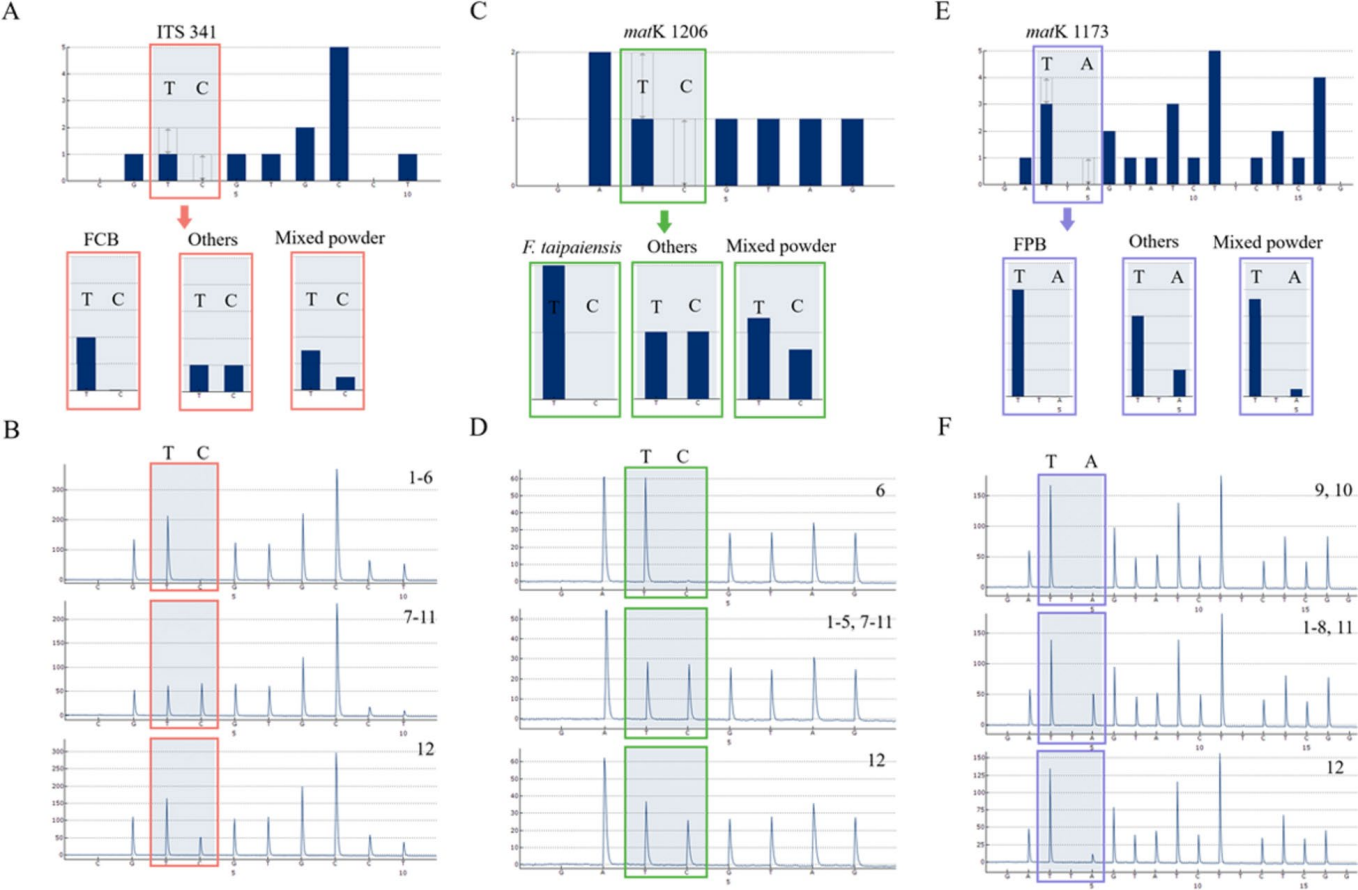
Verification SNPs for FCB, *F. hupehensis* and Fritillariae pallidiflorae bulbus (FPB) at ITS 341, *mat*K 923, and *mat*K 1173 sites. **A, C, E** The standard pyrograms of ITS 341, *mat*K 923, and *mat*K 1173 sites; **B, D, F** The text pyrograms of ITS 341, *mat*K 923, and *mat*K 1173 sites; 1: *F. unibracteata*; 2: *F. unibracteata.var. wabuensis*; 3: *F. cirrhosa*; 4: *F. przewalskii*; 5: *F. delavayi*; 6: *F. taipaiensis*; 7: *F. hupehensis*; 8: *F. ussuriensis*; 9: *F. pallidiflora*; 10: *F. walujewii*; 11: *F. thunbergii*; 12: Mixed powder: It is a 1: 1 mixed powder of six original species and five adulterants of FCB, each of which accounts for 1/11

The validity of the obtained SNPs was verified using pyrosequencing. Among the five specific SNPs, ITS 341 site was used to identify FCB and its adulterants; the genotype of FCB was T, while that of other adulterants was C. When the verified samples had six origins, five adulterants, and a mixed powder of these 11 species (1:1 mixture), the pyrosequencing results showed three types of pyrograms (Fig. 2A and B). Based on this result, ITS 341 site was obtained to specifically distinguish FCB and its adulterants. Among the specific identification sites for the five adulterants of FCB, *mat*K 923, and *mat*K 1173 sites were used to identify *F. hupehensis* and FPB, respectively. At *mat*K 923 site, pyrosequencing was expected to result in an A genotype for *F. hupehensis*, a G genotype for the remaining ten single samples, and both A and G genotypes for the mixed powder of FCB and its adulterants (Fig. 2C and D). At *mat*K 1173 site, the genotype of FPB was A, that of the other nine species was T, and the mixed powder of the 11 species contained both T and A. The verification results for these samples are shown in Fig. 2E and F, 1–12. These results were consistent with the expectations, indicating that the *mat*K 923, and *mat*K 1173 sites were successfully verified. The verification results for the other two SNPs are shown in Supplementary Fig. 1. In subsequent experiment, these five SNPs were combined with Herb-Q to identify the six origins and five adulterants of FCB.

### Establishment of Herb-Q methodology

In the present study, mixtures of different proportions were prepared using powders of FCB and *F. hupehensis*. The linear relationship, LOD, and LOQ of Herb-Q were explored and evaluated for reproducibility and accuracy. The ITS 341 site was randomly chosen as the identification point to complete the methodological study.

#### Linearity, LOD and LOQ determination

**Fig. 3:**
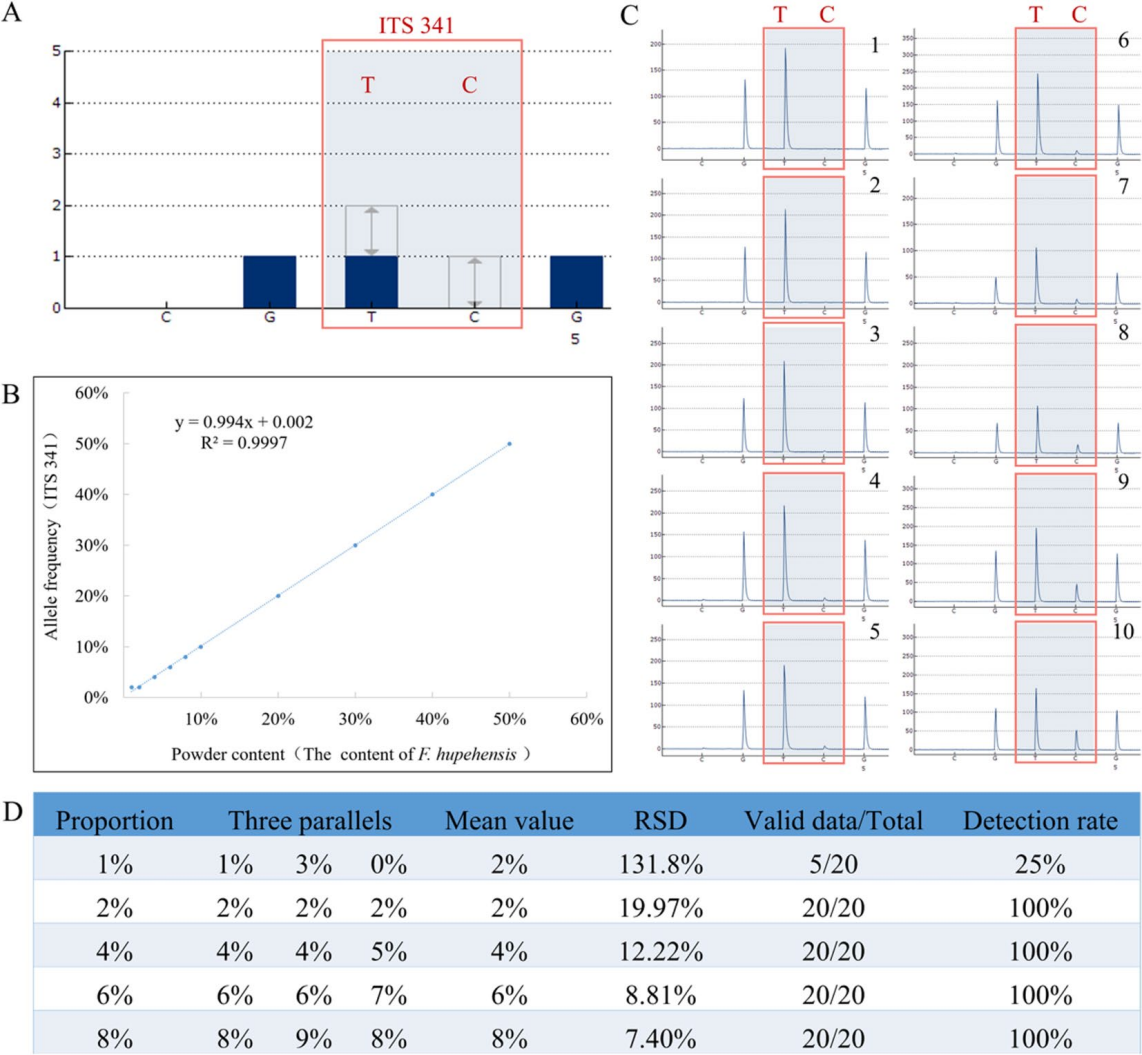
Linear equation of herb molecular quantification (Herb-Q) method at ITS 341 site and its ten proportional pyrograms. **A** The standard pyrograms of ITS 341 site; **B** Linear regression equation of mixed samples of FCB and *F. hupehensis,* with the R^2^ = 0.9997 (≥ 0.99); **C**: The ten proportional pyrograms of mixtures at ITS 341 site; **C1–C10** The ratio of *hupehensis* to FCB is 1, 2 4, 6, 8, 10, 20, 30, 40 and 50% (wt/wt) respectively. **D** LODs and LOQs for *F. hupehensis* adulteration in FCB by Herb-Q

A linear equation was established with the proportion (x) and allelic frequency (y) of *F. hupehensis* (base C) (Fig. 3A). The regression equation was y = 0.994 x + 0.002, and the R^2^ value was 0.9997 (> 0.99), indicating a good linear relationship (Fig. 3B). Accurate allele frequencies were obtained for specific sample proportions (except for 1%). However, the allele frequencies of the samples at 1% were not consistent with the expected values (Fig. 3C; Supplemental Table 7).

The RSD were 131.8% and > 25% when the adulteration rate was 1% in the mixed samples. Hence, the LOQ of the Herb-Q was 2%. The LOD was determined to be 2%, as the stable detection of 2% adulteration in the mixed powder reached 95% (Fig. 3D; Supplementary Table 8).

#### Repeatability and accuracy

To further evaluate the reproducibility and accuracy of Herb-Q, mixed powders were prepared in specific proportions. The average allele frequencies differed by approximately ± 0% or 1%, and the RSDs were all below 25%, showing the good repeatability and accuracy of Herb-Q (Table 1).

**Table 1:** Verification results of quantification for samples with known proportions (n = 6)

Six parallels	Actual proportion		
	15% *F. hupehensis* /85% FCB	25% *F. hupehensis* /75% FCB	35% *F. hupehensis* /65% FCB
1	14/86	25/75	35/65
2	14/86	25/75	34/66
3	15/85	24/76	35/65
4	15/85	25/75	35/65
5	15/85	25/75	36/64
6	15/85	24/76	35/65
Average value (%)	15/85	24/76	35/65
RSD (%)	3.52/0.61	2.09/0.69	1.81/0.97

### Quantitative analysis by Herb-Q

*F. ussuriensis* and *F. thunbergii* have recently been shown to be adulterated or mixed with FCB because of their high market output, low price, and similar bulbs. In this study, FCB, *F. ussuriensis*, and *F. thunbergii* were selected to construct the Herb-Q system.

#### Establishment of the quantitative standard curve

**Fig. 4:**
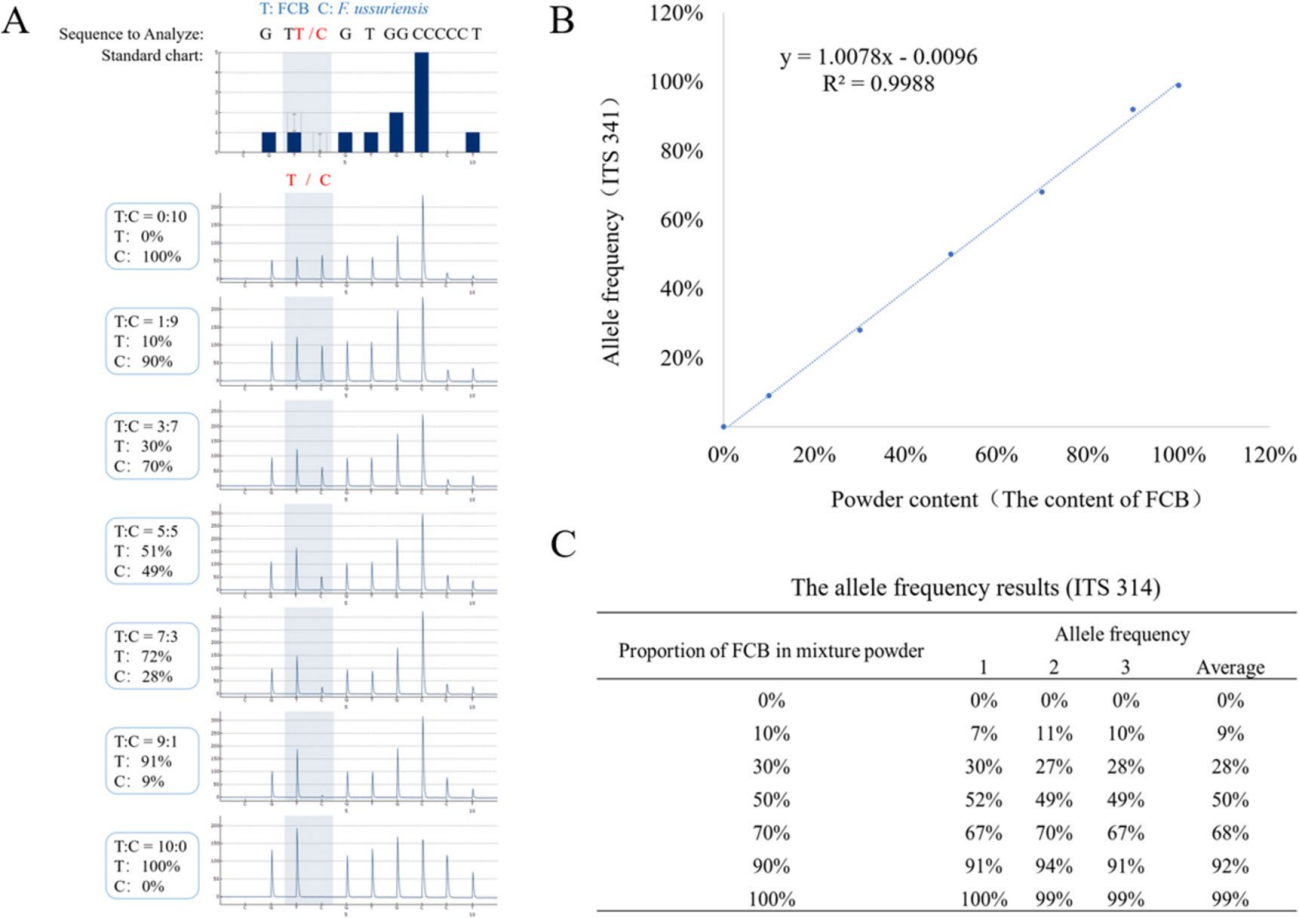
Pyrograms of different proportions at ITS 341 site and the coefficient of determination for linearity. **A** The standard pyrograms of ITS 341 site and the pyrograms of seven mixed powders of FCB and *F. ussuriensis* with the proportions of 0:100, 10:90, 30:70, 50:50, 70:30, 90:10 and 100%:0% (wt/wt); **B** Linear regression equation of mixed samples of FCB and *F. ussuriensis,* with the R^2^ 0.9988; **C** Six allele frequencies and their average values of FCB in mixed powder at ITS 341 site

Mixed powders of FCB and *F. ussuriensis* were randomly selected as samples for a quantitative linear relationship study. The quantitative standard curve of ITS 341 site was established using Herb-Q based on the proportion of the mixed powders and the corresponding allele frequencies. Seven mixing ratios were prepared with the powder of FCB and *F. ussuriensis,* and their corresponding allelic frequencies are shown in the pyrograms (Fig. 4A). A linear equation was established with these seven proportions and the allelic frequency of the base T. The regression equation for the proportion of FCB (x) and allelic frequency of FCB was y = 1.0078 x + 0.0096, and the R^2^ value was 0.9988 (> 0.99), indicating a good linear relationship (Fig. 4B, C). Based on these results, a quantitative standard curve of ITS 341 site of FCB was successfully constructed using Herb-Q. In subsequent applications, the establishment of a quantitative linear relationship provides the basis for constructing a quantitative system.

#### Calculation of quantitative results

**Fig. 5:**
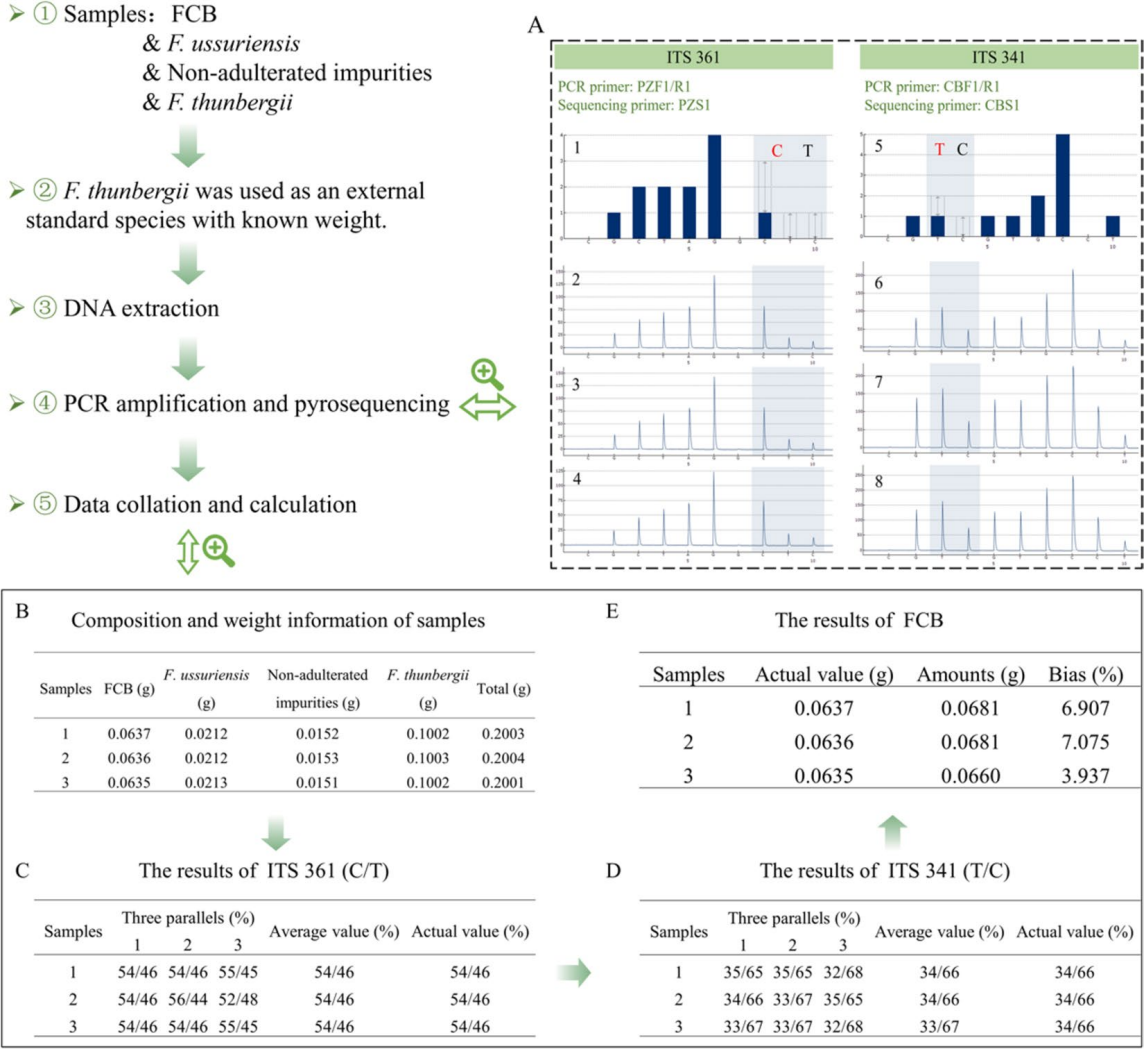
Herb-Q to construct an absolute quantitative process. **A1/A5** The standard pyrograms of ITS 361, and ITS 341 sites; **A2/A6** The text pyrograms of wheat-flour in the samples at ITS 361, and ITS 341 sites; **A3/A7**: The text pyrograms of soil in the samples at ITS 361, and ITS 341 sites; **A4/A8** The text pyrograms of wheat-flour and soil in the samples at ITS 361, and ITS 341 sites; **B** Composition and weight information of three samples; **C** Allele frequency of three samples at ITS 361 site; **D** Allele frequency of three samples at ITS 341 site; **E** Actual value, calculated value and bias of FCB in three samples

To accurately calculate the actual weight of each constituent substance in the presence of non-adulterated impurities, three types of mixed powders were prepared, and the corresponding pyrograms were obtained (Fig. 5A1-8, B). Taking Sample 1 as an example, the weight of *F. thunbergii* was 0.1002 g, and the average allele frequency of base C was 54% at ITS 361 site, which can be deduced to be 0.2003 g of total weight of the mixed powder. Second, the genotype of FCB was T at ITS 341 site, with an average allele frequency of 34%. It can be inferred that the amount of FCB powder in the mixed powder was 0.0681 g, and the bias between it and the actual value was 6.907%, which was within the range of ± 25% (Fig. 5C–E). Similarly, for Samples 2 and 3, the biases between the measured and the actual values were 7.075% and 3.937%, respectively, within the range of ± 25% (Fig. 5C–E). The other results are shown in Supplementary Fig. 2. For non-adulterated species in the mixed powder, Herb-Q successfully quantified FCB and its adulterants with reasonable accuracy.

### Quantitative analysis of cpms by Herb-Q

**Fig. 6:**
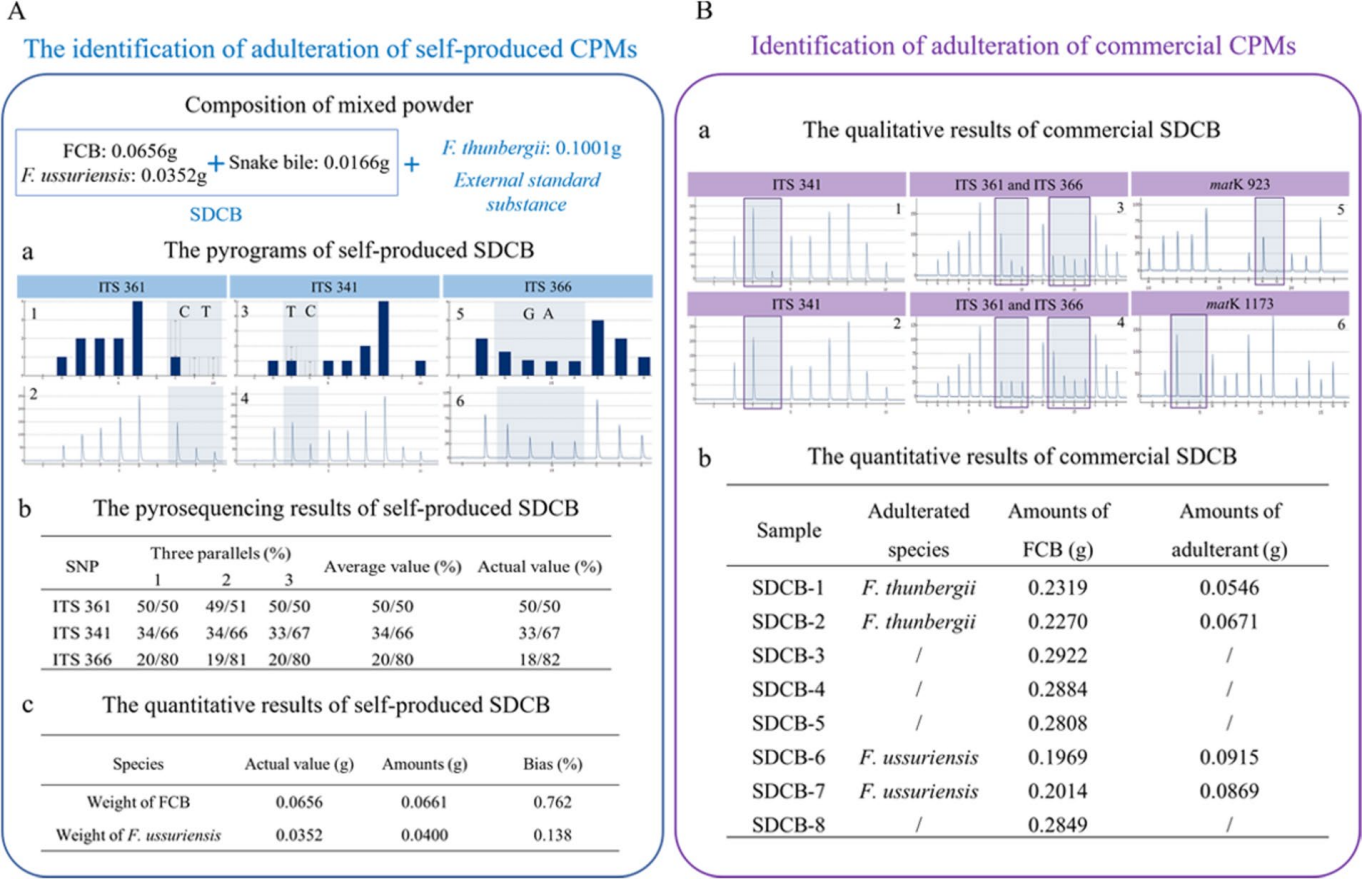
The quantitative identification results of self-produced and commercial Chinese patent medicines (CPMs) by Herb-Q. **A** The figure contains the quantitative identification process of self-produced Shedan Chuanbei capsule (SDCB); a1, a3, a5: The standard pyrograms of SDCB at ITS 361, ITS 341, and ITS 366 sites; a2, a4, a6: The text pyrograms of self-produced SDCB at ITS 361, ITS 341, and ITS 366 sites; b: Pyrosequencing results of self-produced SDCB at ITS 361, ITS 341, and ITS 366 sites; c: Quantitative results of pyrosequencing of self-produced SDCB. **B** The figure contains the quantitative identification process of commercial SDCBs; a1: Qualitative results of pyrosequencing of commercial SDCB 3–5 and 8 at ITS 341 site; a2: Qualitative results of pyrosequencing of commercial SDCB 1–2 and 6–7 at ITS 341 site; a3: Qualitative results of pyrosequencing of commercial SDCB 1–2 at ITS 361, and ITS 366 sites; a4: Qualitative results of pyrosequencing of commercial SDCB 6–7 at ITS 361, and ITS 366 sites; a5, a6: Qualitative results of pyrosequencing of commercial SDCB 1–2 and 6–7 at *mat*K 923, and *mat*K 1173 sites; b: Quantitative results of pyrosequencing of commercial SDCB 1–8

To identify the adulteration of self-produced CPM, the prepared CPM was identified at ITS 341, ITS 361, and ITS 366 sites (Fig. 6A a1–6). At ITS 361 site, *F. thunbergii* had the C genotype and an allele frequency of 50%, and a total weight of 0.2002 g was deduced from its weight of 0.1001 g (Fig. 6A b). At ITS 341 site, FCB had the T genotype, corresponding to an allele frequency of 33%. Based on the total weight, the weight of FCB was determined to be 0.0661 g (Fig. 6A b). At ITS 366 site, the genotype of *F. ussuriensis* was G, the corresponding allele frequency was 20%, and the corresponding weight was 0.0400 g (Fig. 6A b). The biases of FCB and its adulterants were 0.762% and 0.138%, respectively, which are in the range of 25% (Fig. 6A c).

To identify commercial CPMs, ITS 341, ITS 361, ITS 366, *mat*K 923, and *mat*K 1173 sites were used to qualitatively identify eight samples. First, the ITS 341 site was used to determine whether the samples were adulterated. At ITS 341 site, samples 1, 2, 6, and 7 contained bases T and C, while samples 3–5 and 8 only contained base T, indicating that samples 1, 2, 6, and 7 were adulterated, whereas the remaining samples were not (Fig. 6B a1, a2). Second, the types of adulteration in the samples were determined. At ITS 361 site, bases C and T existed in samples 1 and 2, and only base T was present in samples 6 and 7 (Fig. 6B a3). At ITS 366 site, samples 6 and 7 contained bases G and A, while samples 1 and 2 only contained base A (Fig. 6B a4). At *mat*K 923 and *mat*K 1173 sites, samples 1, 2, 6, and 7 did not contain the corresponding genotypes (Fig. 6B a5, a6). As a result, it could be determined that the adulterants in samples 1 and 2 were *F. thunbergii*, the adulterants in samples 6 and 7 were *F. ussuriensis*, and there was no adulteration in samples 3, 5, and 8 *F. hupehensis* was chosen as the external standard substance to be added into the samples for quantitative identification. According to the calculations, samples 1, 2, 6, and 7 contained approximately 0.22 g of FCB, and the weight of the adulterants varied from 0.05 g to 0.10 g, accounting for 9–15% of the total weight. Samples 3, 5, and 8 contained approximately 0.28 g of FCB, which conformed to the Chinese Pharmacopoeia standards (Fig. 6B b; Supplementary Table 9).

## Discussion

FCB is an important herb for treating phlegm and relieving cough, and the export of FCB-derived HMs has increased by 133% from 2015 to 2019 [9]. FCB is often sold as bulbs in the market, but the lack of flower and fruit identification at the time of trade compromises its identification [33]. Adulteration of FCB seriously affects economic trade and the medical industry, especially in the preparation of dietary supplements or cosmetics, where accurate quantification of FCB is essential [34, 35]. Hence, the development of methods for quantitative identification of FCB and its adulterants is valuable. Current methods used to quantify herbal species include quantitative PCR and droplet digital PCR, both of which have been developed based on short sequences with high specificity [23, 36]. However, as FCB is mostly adulterated with closely related species, the interspecific sequences are highly conserved, and it is difficult to screen for suitably specific short fragments. SNPs are single-base variations of species with strong specificity, large number, and wide distribution, which have been widely used in herbs and crops in combination with amplicon resequencing and next-generation sequencing [37–39]. Herb-Q is a rapid and high-throughput molecular method that has demonstrated significant sensitivity and specificity for quantifying allele frequencies in individual patients [26]. In this study, we discovered Herb-Q, a method based on pyrosequencing detecting the fluorescence signal value of specific SNPs, and adding external standard substances to calculate the weight, which can accomplish accurate quantitative detection of mixed species. This method is suitable for the identification of closely related species with high visualization and specificity. It could identify the type and amounts of adulterated species within 4 h and could be easily extended for quantitative detection. Herb-Q provides a means for the quantitative detection of FCB and its adulterants with potential quality control applications.

Eleven species of *Fritillaria* have been recorded in the China Pharmacopoeia, including six original species and five main adulterants of FCB [40]. In total, 170 samples of these species were collected in this study, and species verification was performed using DNA barcoding to ensure sample accuracy. In addition to testing the quantitative validity of Herb-Q, the verified samples were used to prepare mixed powders and self-produced SDCB. SNPs used to identify FCB and its adulterants were determined by comparing 637 ITS, 168 *psb*A-*trn*H, 490 *mat*K and 284 *rbc*L sequences of *Fritillaria*. These sequences were obtained from NCBI and Sanger sequencing, and the number of sequences used ensured the effectiveness of candidate SNPs screening. Various technical parameters such as linearity, LOD, LOQ, reproducibility, and accuracy were evaluated to ensure that Herb-Q could meet the needs of quantitative assays [30]. Quantitative curves were established from multiple mixed powder ratios and allele frequency results. Appropriate external standards were mixed with the samples for quantitative analysis based on the qualitative results. Notably, in this study, the validity of Herb-Q quantitative analysis of CPMs was first verified by the quantitative detection of self-produced samples, and then commercial SDCBs were analyzed. The bias in the results was significantly lower than the reference value (25%) for both mixed powder and CPMs and was also lower than that of other molecular methods reported at present [24].

**Fig. 7:**
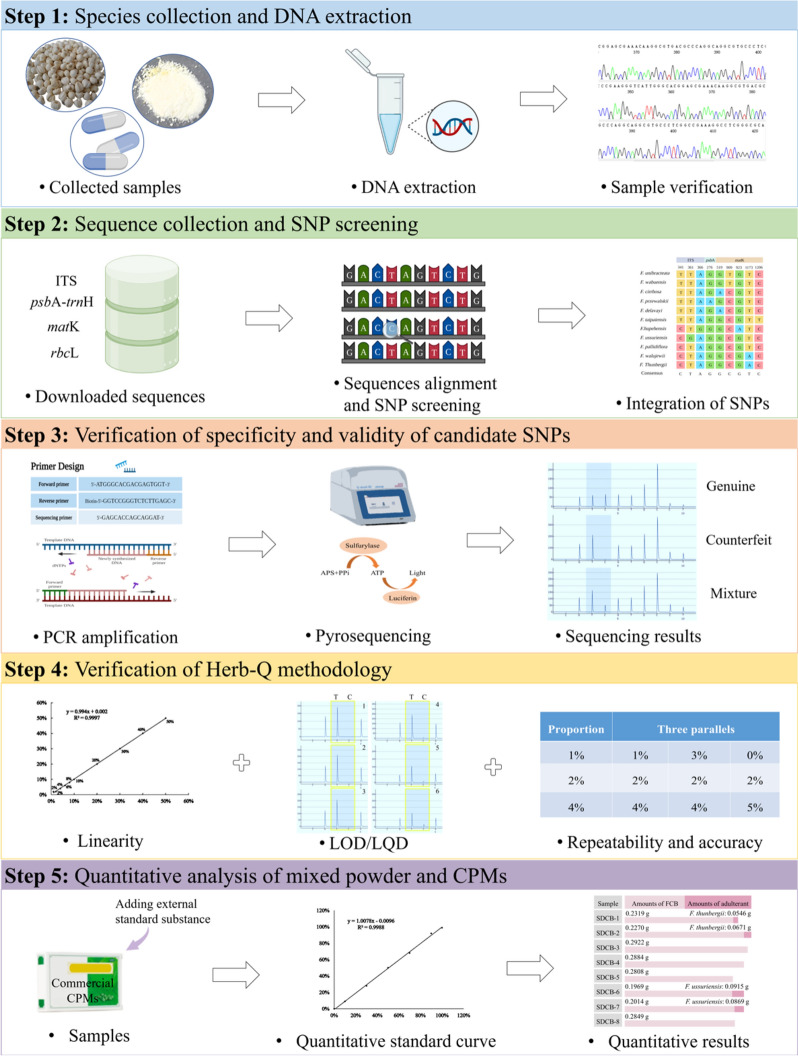
The quantitative identification process of FCB and its adulterants by Herb-Q. Step 1: Species collection and DNA extraction. Step 2: Sequence collection and SNP screening. Step 3: Verification of specificity and validity of candidate SNPs. Step 4: Verification of Herb-Q methodology. Step 5: Quantitative analysis of mixed powder and CPMs. Figures generated with BioRender (https://biorender.com/)

Herb-Q demonstrated accuracy and efficiency in the quantification of FCB and its close relatives and can also be attempted for adulteration identification of other species. Therefore, combined with the research results, the process of Herb-Q accurate species quantification includes five aspects (Fig. 7). Among these, DNA extraction and screening of specific sites are crucial. The entire research process depends on successful DNA extraction. FCB and its CPMs are primarily used as powders in medicine, and DNA extraction is relatively easy. Although it was challenging to identify the species in the processed CPMs, such as decoction and reflux, the DNA sequences of the herbs became fragmented after processing, making it impossible to carry out subsequent experiments [41]. When fragmented DNA serves as a template for PCR, full-length barcodes may not be amplified. The lack of a PCR product may lead to the incorrect conclusion that the DNA template is absent. The use of primer combinations that result in a very small PCR product (100–200 bp) is a better approach for detecting DNA that may be fragmented [42]. Therefore, shortening the length of PCR amplification can be considered to increase the amplification rate of the processed HMs. Although SNP was a base difference between species, strong conservation of species sequences may affect site screening [43]. In this study, FCB and its adulterated species were identified by comparing five SNPs obtained from common DNA barcoding. Among them, two adulterants (*F. walujewii* and *F. pallidiflora*) shared a single identification site at *mat*K 1173 site, because no single SNP was screened. Although specific sites were found in the chloroplast genome sequences, no appropriate sites were identified because of the inability to satisfy the SNP screening principle for Herb-Q. With the publication of additional *Fritillaria* species sequences, identification sites can be continuously optimized and screened. Sequences can be compared and screened in real-time, and this method is expected to be applicable to species that are currently difficult to identify. For the identification of other species, if suitable SNPs cannot be found by current methods, reported reference genomes can be considered, such as the whole genome and resequencing [44].

Overall, Herb-Q is a quantitative method that is easy to operate, visualize, and standardize and provides a new idea for quantitatively identifying mixed powders and CPMs of FCB. This method can complete quantitative detection within 4 h, and the data can be read directly through the image, which can be further applied to other species in the future. In contrast to other quantitative methods such as HPLC/GC, which allow for active ingredient quantification but are only indicative of the herb, Herb-Q can measure the weight of species and represent the characteristics of HMs themselves [45–47]. It is more extensive for the identification of mixed species, and could be used to quantitatively detect adulteration amounts in products, such as Chinese medicinal preparations, health care products, and agricultural products.

## Conclusions

A rapid and accurate Herb-Q was applied to the quantitative detection of adulterants in FCB and its CPMs. Five SNPs were used to identify FCB and its five adulterants, of which the ITS 341 site could discriminate FCB and its main adulterants, and the remaining four SNPs could distinguish the types of each adulterant. A quantitative relationship of Herb-Q was established between the proportion of mixed powders and allele frequency, showing a good linear relationship. Herb-Q was effective and accurate for the quantitative detection of mixed powder and self-made SDCB in the presence of external standard substances, with an average bias of 1.083%. A 9–15% adulteration in eight batches of commodity SDCB was detected using this method. Therefore, the experimental validation of the established Herb-Q assay has confirmed its suitability and high accuracy for quantitative species analysis in FCB and its products. Additionally, it exhibits great potential for wide application in quantifying individual species of other herbs, even their products.

## Supplementary information

Additional file 1

Additional file 2

## Data Availability

The research data generated from this study are included in the article and Additional files.

## Abbreviations

CPMs: Chinese patent medicines
FCB: Fritillariae Cirrhosae Bulbus
FPB: Fritillariae pallidiflorae bulbus
Herb-Q: Herb molecular quantification
HMs: Herbal medicines
LDA: Linear discriminant analysis
LIBS: Laser-induced breakdown spectroscopy
LOD: Limit of detection
LOQ: Limit of quantitation
NIR: Near infrared spectroscopy
PLS: Partial least squares
SNPs: Single nucleotide polymorphisms
SDCB: Shedan Chuanbei capsule
WHO: World Health Organization
