# Supplementary material for: Molecular quantification of fritillariae cirrhosae bulbus and its adulterants
Source: Chin Med. 2024 Oct 8;19:138. doi: 10.1186/s13020-024-01010-z (PMC11460136; doi:10.1186/s13020-024-01010-z)
Supplement: Supplementary file 1 — Additional file 1 [file 13020_2024_1010_MOESM1_ESM.docx]

**Molecular quantification of Fritillariae Cirrhosae Bulbus and its adulterants**


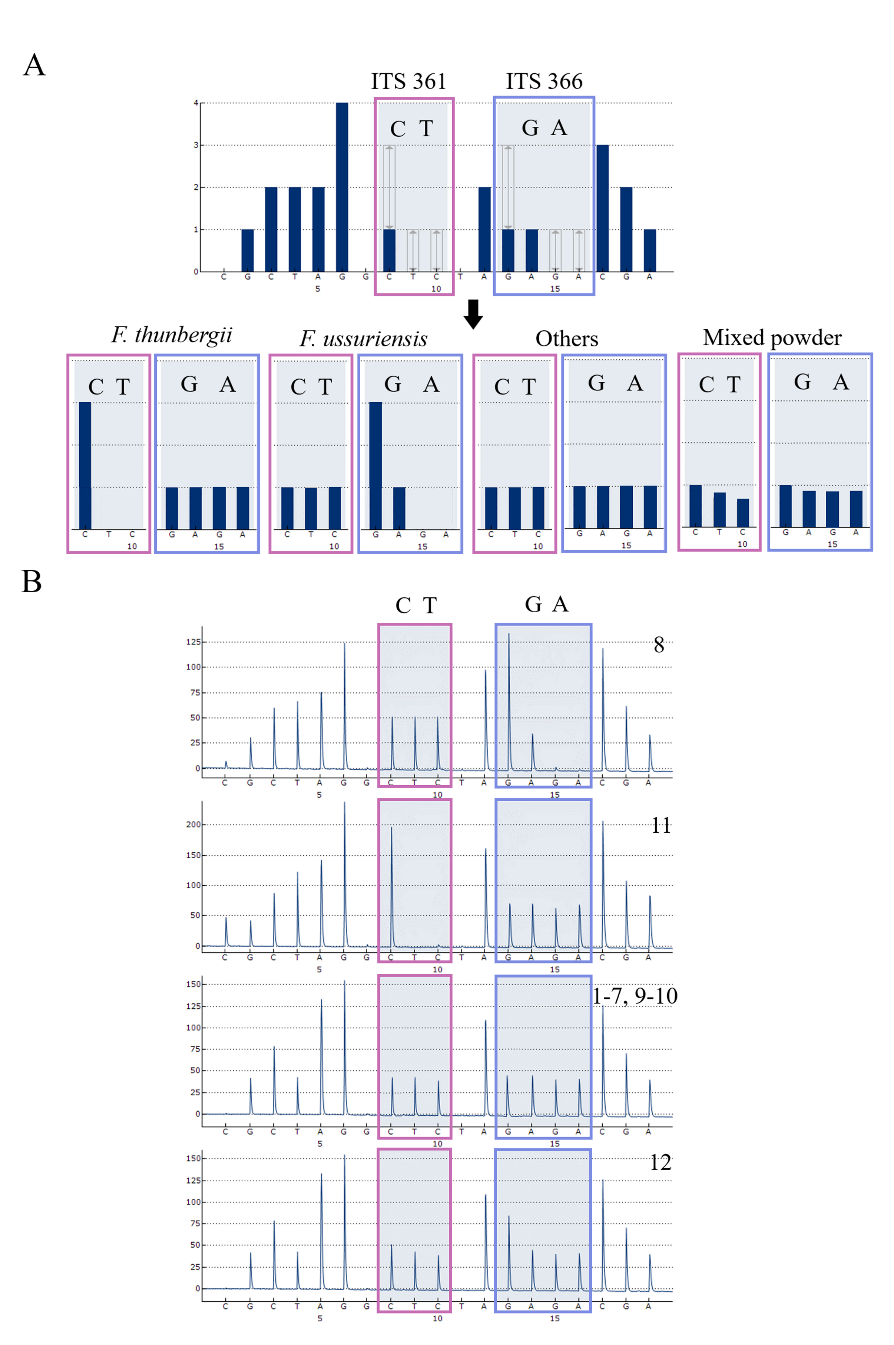


**Supplementary Figure 1**


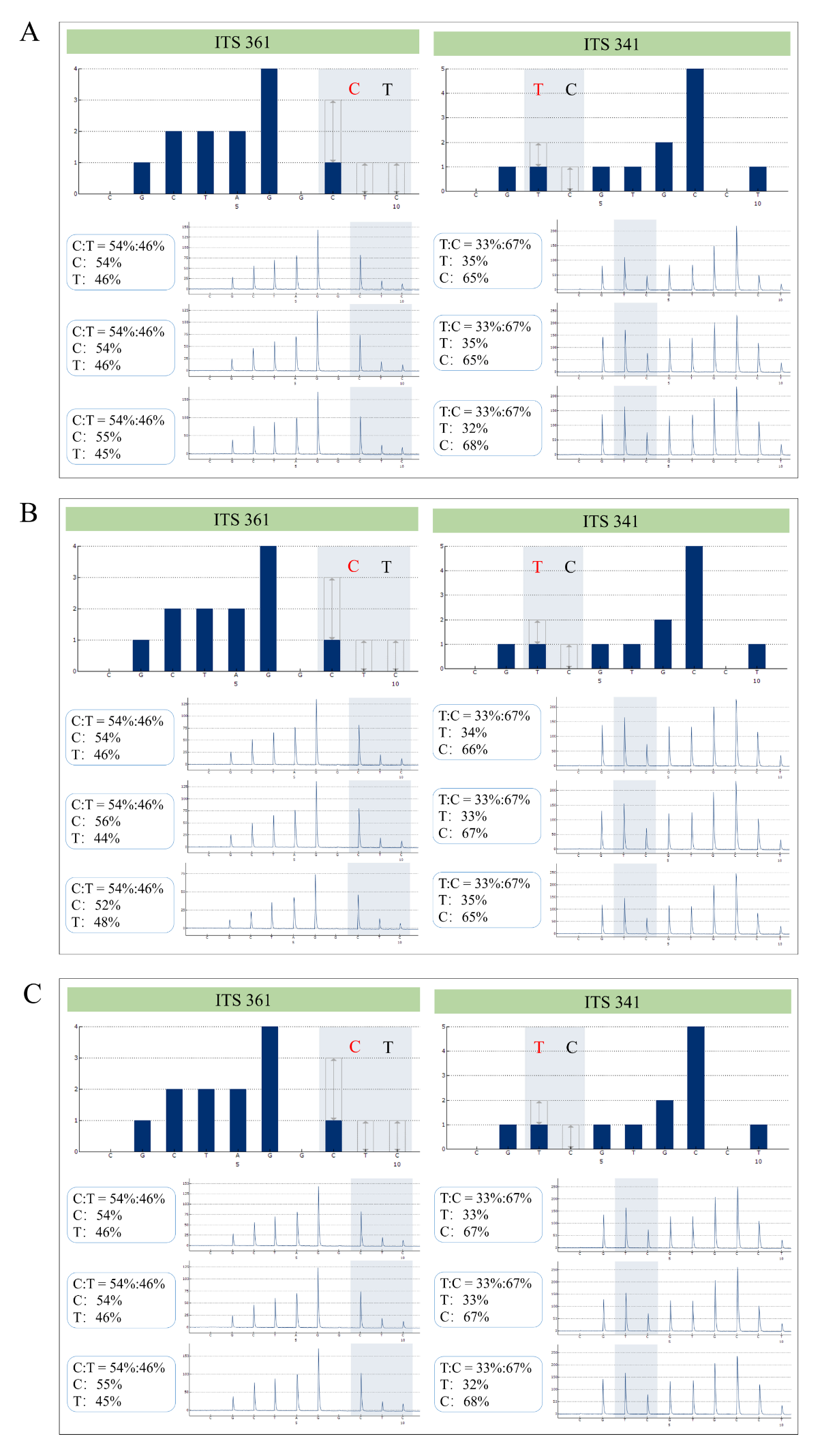


**Supplementary Figure 2**

**Supplementary Figure 1** The verification results of SNPs for ITS 361 and ITS 366 sites. A: The standard pyrograms of ITS 361 and ITS 366 sites; B: The text pyrograms of ITS 361 and ITS 366 sites; 1: *F. unibracteata*; 2: *F. unibracteata.var. wabuensis*; 3: *F. cirrhosa*; 4: *F. przewalskii*; 5: *F. delavayi*; 6: *F. taipaiensis*; 7: *F. hupehensis*; 8: *F. ussuriensis*; 9: *F. pallidiflora*; 10: *F. walujewii*; 11: *F. thunbergii*; 12: Mixed powder: It is a 1: 1 mixed powder of six original species and five adulterants of FCB, each of which accounts for 1/11.

**Supplementary Figure 2** Herb-Q to construct an absolute quantitative process. A: The text pyrograms of wheat-flour in the samples at ITS 361 and ITS 341 sites; B: T The text pyrograms of soil in the samples at ITS 361 and ITS 341 sites; C: The text pyrograms of wheat-flour and soil in the samples at ITS 361 and ITS 341 sites.
