# Supplementary material for: Molecular quantification of fritillariae cirrhosae bulbus and its adulterants
Source: Chin Med. 2024 Oct 8;19:138. doi: 10.1186/s13020-024-01010-z (PMC11460136; doi:10.1186/s13020-024-01010-z)
Supplement: Supplementary file 2 — Additional file 2 [file 13020_2024_1010_MOESM2_ESM.docx]

| **Title** | Molecular quantification of Fritillariae Cirrhosae Bulbus and its adulterants |
| --- | --- |
| **Author information** | Ziyi Liu^a,b,†^, Yifei Pei^a,†^, Tiezhu Chen^b^, Zemin Yang^a^, Wenjun Jiang^a^, Xue Feng^a,^*, and Xiwen Li^a,^* |
|  | ^†^ These authors contributed equally to this article |
| **Corresponding author** | Xiwen Li*, Xue Feng* |
| **E-mail address of corresponding author** | xwli@icmm.ac.cn; xfeng0413@icmm.ac.cn; |
| **Present/permanent address** | ^a^ Institute of Chinese Materia Medica, China Academy of Chinese Medical Sciences, Beijing 100700, China; ^b^ Sichuan Provincial Key Laboratory of Quality and Innovation Research of Chinese Materia Medica, Sichuan Academy of Traditional Chinese Medicine Sciences, Chengdu 610041, China |
| **Contents** | **Supplementary Table 1**  The sampling information of Fritillariae Cirrhosae Bulbus and its adulterated species |
|  | **Supplementary Table 2** PCR and sequencing primers for pyrosequencing |
|  | **Supplementary Table 3a** Collection of ITS sequences of *Fritillaria* |
|  | **Supplementary Table 3b** Collection of *psb*A-*trn*H sequences of *Fritillaria* |
|  | **Supplementary Table 3c** Collection of *rbcL* sequences of *Fritillaria* |
|  | **Supplementary Table 3d** Collection of *matK* sequences of *Fritillaria* |
|  | **Supplementary Table 4**  Candidate SNPs in Fritillariae Cirrhosae Bulbus ITS and *mat*K sequences |
|  | **Supplementary Table 5** Candidate SNPs in ITS and *mat*K sequences for individual adulterated species |
|  | **Supplementary Table 6** Detailed BLAST results with the score being max and total |
|  | **Supplementary Table 7** The raw data of all proportions for Linearity (n=3) |
|  | **Supplementary Table 8** The raw data of all proportions for LOD/LOQ (n=20) |
|  | **Supplementary Table 9a**  The results of commercial CPMs in *mat*K 923 |
|  | **Supplementary Table 9b** The results of commercial CPMs in ITS 341 |
|  | **Supplementary Table 9c** The results of commercial CPMs in ITS 366 |

Supplementary Table 1 The sampling information of Fritillariae Cirrhosae Bulbus and its adulterated species

| Sample No. | Latin Name of Original Species | English name of Medicinal materials | Sample type | Collection Site | Longitude | Latitude | Altitude (m) |
| --- | --- | --- | --- | --- | --- | --- | --- |
| JY-01 | *Fritillaria cirrhosa* | Fritillariae cirrhosae bulbus | Original Plant | Haidong, Qinghai Province | 102°00' | 37°00' | 2900 |
| JY-02 | *Fritillaria cirrhosa* | Fritillariae cirrhosae bulbus | Original Plant | Haidong, Qinghai Province | 102°00' | 37°00' | 2900 |
| JY-03 | *Fritillaria cirrhosa* | Fritillariae cirrhosae bulbus | Original Plant | Haidong, Qinghai Province | 102°00' | 37°00' | 2900 |
| JY-04 | *Fritillaria cirrhosa* | Fritillariae cirrhosae bulbus | Original Plant | Haidong, Qinghai Province | 102°00' | 37°00' | 2900 |
| JY-05 | *Fritillaria cirrhosa* | Fritillariae cirrhosae bulbus | Original Plant | Haidong, Qinghai Province | 102°00' | 37°00' | 2900 |
| JY-06 | *Fritillaria cirrhosa* | Fritillariae cirrhosae bulbus | Original Plant | Haidong, Qinghai Province | 102°00' | 37°00' | 2900 |
| JY-07 | *Fritillaria cirrhosa* | Fritillariae cirrhosae bulbus | Original Plant | Haidong, Qinghai Province | 102°00' | 37°00' | 2900 |
| JY-08 | *Fritillaria cirrhosa* | Fritillariae cirrhosae bulbus | Original Plant | Haidong, Qinghai Province | 102°00' | 37°00' | 2900 |
| JY-09 | *Fritillaria cirrhosa* | Fritillariae cirrhosae bulbus | Original Plant | Haidong, Qinghai Province | 102°00' | 37°00' | 2900 |
| JY-10 | *Fritillaria cirrhosa* | Fritillariae cirrhosae bulbus | Original Plant | Haidong, Qinghai Province | 102°00' | 37°00' | 2900 |
| JY-11 | *Fritillaria cirrhosa* | Fritillariae cirrhosae bulbus | Original Plant | Ganzi, Sichuan Province | 101°58' | 30°06' | 3050 |
| JY-12 | *Fritillaria cirrhosa* | Fritillariae cirrhosae bulbus | Original Plant | Ganzi, Sichuan Province | 101°58' | 30°06' | 3050 |
| JY-13 | *Fritillaria cirrhosa* | Fritillariae cirrhosae bulbus | Original Plant | Ganzi, Sichuan Province | 101°58' | 30°06' | 3050 |
| JY-14 | *Fritillaria cirrhosa* | Fritillariae cirrhosae bulbus | Original Plant | Ganzi, Sichuan Province | 101°58' | 30°06' | 3050 |
| JY-15 | *Fritillaria cirrhosa* | Fritillariae cirrhosae bulbus | Original Plant | Ganzi, Sichuan Province | 101°58' | 30°06' | 3050 |
| JY-16 | *Fritillaria cirrhosa* | Fritillariae cirrhosae bulbus | Original Plant | Ganzi, Sichuan Province | 101°58' | 30°06' | 3050 |
| JY-17 | *Fritillaria cirrhosa* | Fritillariae cirrhosae bulbus | Original Plant | Ganzi, Sichuan Province | 101°58' | 30°06' | 3050 |
| JY-18 | *Fritillaria cirrhosa* | Fritillariae cirrhosae bulbus | Original Plant | Ganzi, Sichuan Province | 101°58' | 30°06' | 3050 |
| JY-19 | *Fritillaria cirrhosa* | Fritillariae cirrhosae bulbus | Original Plant | Ganzi, Sichuan Province | 101°58' | 30°06' | 3050 |
| JY-20 | *Fritillaria cirrhosa* | Fritillariae cirrhosae bulbus | Original Plant | Ganzi, Sichuan Province | 101°58' | 30°06' | 3050 |
| AZ-01 | *Fritillaria unibracteata* | Fritillariae cirrhosae bulbus | Original Plant | Haidong, Qinghai Province | 102°00' | 37°00' | 2900 |
| AZ-02 | *Fritillaria unibracteata* | Fritillariae cirrhosae bulbus | Original Plant | Haidong, Qinghai Province | 102°00' | 37°00' | 2900 |
| AZ-03 | *Fritillaria unibracteata* | Fritillariae cirrhosae bulbus | Original Plant | Haidong, Qinghai Province | 102°00' | 37°00' | 2900 |
| AZ-04 | *Fritillaria unibracteata* | Fritillariae cirrhosae bulbus | Original Plant | Haidong, Qinghai Province | 102°00' | 37°00' | 2900 |
| AZ-05 | *Fritillaria unibracteata* | Fritillariae cirrhosae bulbus | Original Plant | Haidong, Qinghai Province | 102°00' | 37°00' | 2900 |
| AZ-06 | *Fritillaria unibracteata* | Fritillariae cirrhosae bulbus | Original Plant | Haidong, Qinghai Province | 102°00' | 37°00' | 2900 |
| AZ-07 | *Fritillaria unibracteata* | Fritillariae cirrhosae bulbus | Original Plant | Haidong, Qinghai Province | 102°00' | 37°00' | 2900 |
| AZ-08 | *Fritillaria unibracteata* | Fritillariae cirrhosae bulbus | Original Plant | Haidong, Qinghai Province | 102°00' | 37°00' | 2900 |
| AZ-09 | *Fritillaria unibracteata* | Fritillariae cirrhosae bulbus | Original Plant | Haidong, Qinghai Province | 102°00' | 37°00' | 2900 |
| AZ-10 | *Fritillaria unibracteata* | Fritillariae cirrhosae bulbus | Original Plant | Haidong, Qinghai Province | 102°00' | 37°00' | 2900 |
| AZ-11 | *Fritillaria unibracteata* | Fritillariae cirrhosae bulbus | Original Plant | Aba, Sichuan Province | 103°63' | 32°63' | 3542 |
| AZ-12 | *Fritillaria unibracteata* | Fritillariae cirrhosae bulbus | Original Plant | Aba, Sichuan Province | 103°63' | 32°63' | 3542 |
| AZ-13 | *Fritillaria unibracteata* | Fritillariae cirrhosae bulbus | Original Plant | Aba, Sichuan Province | 103°63' | 32°63' | 3542 |
| AZ-14 | *Fritillaria unibracteata* | Fritillariae cirrhosae bulbus | Original Plant | Aba, Sichuan Province | 103°63' | 32°63' | 3542 |
| AZ-15 | *Fritillaria unibracteata* | Fritillariae cirrhosae bulbus | Original Plant | Aba, Sichuan Province | 103°63' | 32°63' | 3542 |
| AZ-16 | *Fritillaria unibracteata* | Fritillariae cirrhosae bulbus | Original Plant | Aba, Sichuan Province | 103°63' | 32°63' | 3542 |
| AZ-17 | *Fritillaria unibracteata* | Fritillariae cirrhosae bulbus | Original Plant | Aba, Sichuan Province | 103°63' | 32°63' | 3542 |
| AZ-18 | *Fritillaria unibracteata* | Fritillariae cirrhosae bulbus | Original Plant | Aba, Sichuan Province | 103°63' | 32°63' | 3542 |
| AZ-19 | *Fritillaria unibracteata* | Fritillariae cirrhosae bulbus | Original Plant | Aba, Sichuan Province | 103°63' | 32°63' | 3542 |
| AZ-20 | *Fritillaria unibracteata* | Fritillariae cirrhosae bulbus | Original Plant | Aba, Sichuan Province | 103°63' | 32°63' | 3542 |
| WB-01 | *Fritillaria unibracteata.var. wabuensis* | Fritillariae cirrhosae bulbus | Original Plant | Haidong, Qinghai Province | 102°00' | 37°00' | 2900 |
| WB-02 | *Fritillaria unibracteata.var. wabuensis* | Fritillariae cirrhosae bulbus | Original Plant | Haidong, Qinghai Province | 102°00' | 37°00' | 2900 |
| WB-03 | *Fritillaria unibracteata.var. wabuensis* | Fritillariae cirrhosae bulbus | Original Plant | Haidong, Qinghai Province | 102°00' | 37°00' | 2900 |
| WB-04 | *Fritillaria unibracteata.var. wabuensis* | Fritillariae cirrhosae bulbus | Original Plant | Haidong, Qinghai Province | 102°00' | 37°00' | 2900 |
| WB-05 | *Fritillaria unibracteata.var. wabuensis* | Fritillariae cirrhosae bulbus | Original Plant | Haidong, Qinghai Province | 102°00' | 37°00' | 2900 |
| WB-06 | *Fritillaria unibracteata.var. wabuensis* | Fritillariae cirrhosae bulbus | Original Plant | Haidong, Qinghai Province | 102°00' | 37°00' | 2900 |
| WB-07 | *Fritillaria unibracteata.var. wabuensis* | Fritillariae cirrhosae bulbus | Original Plant | Haidong, Qinghai Province | 102°00' | 37°00' | 2900 |
| WB-08 | *Fritillaria unibracteata.var. wabuensis* | Fritillariae cirrhosae bulbus | Original Plant | Haidong, Qinghai Province | 102°00' | 37°00' | 2900 |
| WB-09 | *Fritillaria unibracteata.var. wabuensis* | Fritillariae cirrhosae bulbus | Original Plant | Haidong, Qinghai Province | 102°00' | 37°00' | 2900 |
| WB-10 | *Fritillaria unibracteata.var. wabuensis* | Fritillariae cirrhosae bulbus | Original Plant | Haidong, Qinghai Province | 102°00' | 37°00' | 2900 |
| WB-11 | *Fritillaria unibracteata.var. wabuensis* | Fritillariae cirrhosae bulbus | Original Plant | Aba, Sichuan Province | 103°63' | 32°63' | 3542 |
| WB-12 | *Fritillaria unibracteata.var. wabuensis* | Fritillariae cirrhosae bulbus | Original Plant | Aba, Sichuan Province | 103°63' | 32°63' | 3542 |
| WB-13 | *Fritillaria unibracteata.var. wabuensis* | Fritillariae cirrhosae bulbus | Original Plant | Aba, Sichuan Province | 103°63' | 32°63' | 3542 |
| WB-14 | *Fritillaria unibracteata.var. wabuensis* | Fritillariae cirrhosae bulbus | Original Plant | Aba, Sichuan Province | 103°63' | 32°63' | 3542 |
| WB-15 | *Fritillaria unibracteata.var. wabuensis* | Fritillariae cirrhosae bulbus | Original Plant | Aba, Sichuan Province | 103°63' | 32°63' | 3542 |
| WB-16 | *Fritillaria unibracteata.var. wabuensis* | Fritillariae cirrhosae bulbus | Original Plant | Aba, Sichuan Province | 103°63' | 32°63' | 3542 |
| WB-17 | *Fritillaria unibracteata.var. wabuensis* | Fritillariae cirrhosae bulbus | Original Plant | Aba, Sichuan Province | 103°63' | 32°63' | 3542 |
| WB-18 | *Fritillaria unibracteata.var. wabuensis* | Fritillariae cirrhosae bulbus | Original Plant | Aba, Sichuan Province | 103°63' | 32°63' | 3542 |
| WB-19 | *Fritillaria unibracteata.var. wabuensis* | Fritillariae cirrhosae bulbus | Original Plant | Aba, Sichuan Province | 103°63' | 32°63' | 3542 |
| WB-20 | *Fritillaria unibracteata.var. wabuensis* | Fritillariae cirrhosae bulbus | Original Plant | Aba, Sichuan Province | 103°63' | 32°63' | 3542 |
| GS-01 | *Fritillaria przewalskii* | Fritillariae cirrhosae bulbus | Original Plant | Haidong, Qinghai Province | 102°00' | 37°00' | 2900 |
| GS-02 | *Fritillaria przewalskii* | Fritillariae cirrhosae bulbus | Original Plant | Haidong, Qinghai Province | 102°00' | 37°00' | 2900 |
| GS-03 | *Fritillaria przewalskii* | Fritillariae cirrhosae bulbus | Original Plant | Haidong, Qinghai Province | 102°00' | 37°00' | 2900 |
| GS-04 | *Fritillaria przewalskii* | Fritillariae cirrhosae bulbus | Original Plant | Haidong, Qinghai Province | 102°00' | 37°00' | 2900 |
| GS-05 | *Fritillaria przewalskii* | Fritillariae cirrhosae bulbus | Original Plant | Haidong, Qinghai Province | 102°00' | 37°00' | 2900 |
| GS-06 | *Fritillaria przewalskii* | Fritillariae cirrhosae bulbus | Original Plant | Haidong, Qinghai Province | 102°00' | 37°00' | 2900 |
| GS-07 | *Fritillaria przewalskii* | Fritillariae cirrhosae bulbus | Original Plant | Haidong, Qinghai Province | 102°00' | 37°00' | 2900 |
| GS-08 | *Fritillaria przewalskii* | Fritillariae cirrhosae bulbus | Original Plant | Haidong, Qinghai Province | 102°00' | 37°00' | 2900 |
| GS-09 | *Fritillaria przewalskii* | Fritillariae cirrhosae bulbus | Original Plant | Haidong, Qinghai Province | 102°00' | 37°00' | 2900 |
| GS-10 | *Fritillaria przewalskii* | Fritillariae cirrhosae bulbus | Original Plant | Haidong, Qinghai Province | 102°00' | 37°00' | 2900 |
| GS-11 | *Fritillaria przewalskii* | Fritillariae cirrhosae bulbus | Original Plant | Tianshui, Gansu Province | 120°28' | 28°10' | 1620 |
| GS-12 | *Fritillaria przewalskii* | Fritillariae cirrhosae bulbus | Original Plant | Tianshui, Gansu Province | 120°28' | 28°10' | 1620 |
| GS-13 | *Fritillaria przewalskii* | Fritillariae cirrhosae bulbus | Original Plant | Tianshui, Gansu Province | 120°28' | 28°10' | 1620 |
| GS-14 | *Fritillaria przewalskii* | Fritillariae cirrhosae bulbus | Original Plant | Tianshui, Gansu Province | 120°28' | 28°10' | 1620 |
| GS-15 | *Fritillaria przewalskii* | Fritillariae cirrhosae bulbus | Original Plant | Tianshui, Gansu Province | 120°28' | 28°10' | 1620 |
| GS-16 | *Fritillaria przewalskii* | Fritillariae cirrhosae bulbus | Original Plant | Tianshui, Gansu Province | 120°28' | 28°10' | 1620 |
| GS-17 | *Fritillaria przewalskii* | Fritillariae cirrhosae bulbus | Original Plant | Tianshui, Gansu Province | 120°28' | 28°10' | 1620 |
| GS-18 | *Fritillaria przewalskii* | Fritillariae cirrhosae bulbus | Original Plant | Tianshui, Gansu Province | 120°28' | 28°10' | 1620 |
| GS-19 | *Fritillaria przewalskii* | Fritillariae cirrhosae bulbus | Original Plant | Tianshui, Gansu Province | 120°28' | 28°10' | 1620 |
| GS-20 | *Fritillaria przewalskii* | Fritillariae cirrhosae bulbus | Original Plant | Tianshui, Gansu Province | 120°28' | 28°10' | 1620 |
| SS-01 | *Fritillaria delavayi* | Fritillariae cirrhosae bulbus | Original Plant | Haidong, Qinghai Province | 102°00' | 37°00' | 2900 |
| SS-02 | *Fritillaria delavayi* | Fritillariae cirrhosae bulbus | Original Plant | Haidong, Qinghai Province | 102°00' | 37°00' | 2900 |
| SS-03 | *Fritillaria delavayi* | Fritillariae cirrhosae bulbus | Original Plant | Haidong, Qinghai Province | 102°00' | 37°00' | 2900 |
| SS-04 | *Fritillaria delavayi* | Fritillariae cirrhosae bulbus | Original Plant | Haidong, Qinghai Province | 102°00' | 37°00' | 2900 |
| SS-05 | *Fritillaria delavayi* | Fritillariae cirrhosae bulbus | Original Plant | Haidong, Qinghai Province | 102°00' | 37°00' | 2900 |
| SS-06 | *Fritillaria delavayi* | Fritillariae cirrhosae bulbus | Original Plant | Haidong, Qinghai Province | 102°00' | 37°00' | 2900 |
| SS-07 | *Fritillaria delavayi* | Fritillariae cirrhosae bulbus | Original Plant | Haidong, Qinghai Province | 102°00' | 37°00' | 2900 |
| SS-08 | *Fritillaria delavayi* | Fritillariae cirrhosae bulbus | Original Plant | Haidong, Qinghai Province | 102°00' | 37°00' | 2900 |
| SS-09 | *Fritillaria delavayi* | Fritillariae cirrhosae bulbus | Original Plant | Haidong, Qinghai Province | 102°00' | 37°00' | 2900 |
| SS-10 | *Fritillaria delavayi* | Fritillariae cirrhosae bulbus | Original Plant | Haidong, Qinghai Province | 102°00' | 37°00' | 2900 |
| SS-11 | *Fritillaria delavayi* | Fritillariae cirrhosae bulbus | Original Plant | Xianggelila,Yunnan Province | 99°30' | 27°44' | 3678 |
| SS-12 | *Fritillaria delavayi* | Fritillariae cirrhosae bulbus | Original Plant | Xianggelila,Yunnan Province | 99°30' | 27°44' | 3678 |
| SS-13 | *Fritillaria delavayi* | Fritillariae cirrhosae bulbus | Original Plant | Xianggelila,Yunnan Province | 99°30' | 27°44' | 3678 |
| SS-14 | *Fritillaria delavayi* | Fritillariae cirrhosae bulbus | Original Plant | Xianggelila,Yunnan Province | 99°30' | 27°44' | 3678 |
| SS-15 | *Fritillaria delavayi* | Fritillariae cirrhosae bulbus | Original Plant | Xianggelila,Yunnan Province | 99°30' | 27°44' | 3678 |
| SS-16 | *Fritillaria delavayi* | Fritillariae cirrhosae bulbus | Original Plant | Xianggelila,Yunnan Province | 99°30' | 27°44' | 3678 |
| SS-17 | *Fritillaria delavayi* | Fritillariae cirrhosae bulbus | Original Plant | Xianggelila,Yunnan Province | 99°30' | 27°44' | 3678 |
| SS-18 | *Fritillaria delavayi* | Fritillariae cirrhosae bulbus | Original Plant | Xianggelila,Yunnan Province | 99°30' | 27°44' | 3678 |
| SS-19 | *Fritillaria delavayi* | Fritillariae cirrhosae bulbus | Original Plant | Xianggelila,Yunnan Province | 99°30' | 27°44' | 3678 |
| SS-20 | *Fritillaria delavayi* | Fritillariae cirrhosae bulbus | Original Plant | Xianggelila,Yunnan Province | 99°30' | 27°44' | 3678 |
| TB-01 | *Fritillaria taipaiensis* | Fritillariae cirrhosae bulbus | Original Plant | Haidong, Qinghai Province | 102°00' | 37°00' | 2900 |
| TB-02 | *Fritillaria taipaiensis* | Fritillariae cirrhosae bulbus | Original Plant | Haidong, Qinghai Province | 102°00' | 37°00' | 2900 |
| TB-03 | *Fritillaria taipaiensis* | Fritillariae cirrhosae bulbus | Original Plant | Haidong, Qinghai Province | 102°00' | 37°00' | 2900 |
| TB-04 | *Fritillaria taipaiensis* | Fritillariae cirrhosae bulbus | Original Plant | Haidong, Qinghai Province | 102°00' | 37°00' | 2900 |
| TB-05 | *Fritillaria taipaiensis* | Fritillariae cirrhosae bulbus | Original Plant | Haidong, Qinghai Province | 102°00' | 37°00' | 2900 |
| TB-06 | *Fritillaria taipaiensis* | Fritillariae cirrhosae bulbus | Original Plant | Haidong, Qinghai Province | 102°00' | 37°00' | 2900 |
| TB-07 | *Fritillaria taipaiensis* | Fritillariae cirrhosae bulbus | Original Plant | Haidong, Qinghai Province | 102°00' | 37°00' | 2900 |
| TB-08 | *Fritillaria taipaiensis* | Fritillariae cirrhosae bulbus | Original Plant | Haidong, Qinghai Province | 102°00' | 37°00' | 2900 |
| TB-09 | *Fritillaria taipaiensis* | Fritillariae cirrhosae bulbus | Original Plant | Haidong, Qinghai Province | 102°00' | 37°00' | 2900 |
| TB-10 | *Fritillaria taipaiensis* | Fritillariae cirrhosae bulbus | Original Plant | Haidong, Qinghai Province | 102°00' | 37°00' | 2900 |
| TB-11 | *Fritillaria taipaiensis* | Fritillariae cirrhosae bulbus | Original Plant | Baoji, Shanxi Province | 107°81' | 33°58' | 2819 |
| TB-12 | *Fritillaria taipaiensis* | Fritillariae cirrhosae bulbus | Original Plant | Baoji, Shanxi Province | 107°81' | 33°58' | 2819 |
| TB-13 | *Fritillaria taipaiensis* | Fritillariae cirrhosae bulbus | Original Plant | Baoji, Shanxi Province | 107°81' | 33°58' | 2819 |
| TB-14 | *Fritillaria taipaiensis* | Fritillariae cirrhosae bulbus | Original Plant | Baoji, Shanxi Province | 107°81' | 33°58' | 2819 |
| TB-15 | *Fritillaria taipaiensis* | Fritillariae cirrhosae bulbus | Original Plant | Baoji, Shanxi Province | 107°81' | 33°58' | 2819 |
| TB-16 | *Fritillaria taipaiensis* | Fritillariae cirrhosae bulbus | Original Plant | Baoji, Shanxi Province | 107°81' | 33°58' | 2819 |
| TB-17 | *Fritillaria taipaiensis* | Fritillariae cirrhosae bulbus | Original Plant | Baoji, Shanxi Province | 107°81' | 33°58' | 2819 |
| TB-18 | *Fritillaria taipaiensis* | Fritillariae cirrhosae bulbus | Original Plant | Baoji, Shanxi Province | 107°81' | 33°58' | 2819 |
| TB-19 | *Fritillaria taipaiensis* | Fritillariae cirrhosae bulbus | Original Plant | Baoji, Shanxi Province | 107°81' | 33°58' | 2819 |
| TB-20 | *Fritillaria taipaiensis* | Fritillariae cirrhosae bulbus | Original Plant | Baoji, Shanxi Province | 107°81' | 33°58' | 2819 |
| HB-01 | *Fritillaria hupehensis* | Fritillariae hupehensis bulbus | Original Plant | Wuxi, Chongqing City | 109°34' | 31°23' | 1596 |
| HB-02 | *Fritillaria hupehensis* | Fritillariae hupehensis bulbus | Original Plant | Wuxi, Chongqing City | 109°34' | 31°23' | 1596 |
| HB-03 | *Fritillaria hupehensis* | Fritillariae hupehensis bulbus | Original Plant | Wuxi, Chongqing City | 109°34' | 31°23' | 1596 |
| HB-04 | *Fritillaria hupehensis* | Fritillariae hupehensis bulbus | Original Plant | Wuxi, Chongqing City | 109°34' | 31°23' | 1596 |
| HB-05 | *Fritillaria hupehensis* | Fritillariae hupehensis bulbus | Original Plant | Wuxi, Chongqing City | 109°34' | 31°23' | 1596 |
| HB-06 | *Fritillaria hupehensis* | Fritillariae hupehensis bulbus | Original Plant | Wuxi, Chongqing City | 109°34' | 31°23' | 1596 |
| HB-07 | *Fritillaria hupehensis* | Fritillariae hupehensis bulbus | Original Plant | Wuxi, Chongqing City | 109°34' | 31°23' | 1596 |
| HB-08 | *Fritillaria hupehensis* | Fritillariae hupehensis bulbus | Original Plant | Wuxi, Chongqing City | 109°34' | 31°23' | 1596 |
| HB-09 | *Fritillaria hupehensis* | Fritillariae hupehensis bulbus | Original Plant | Wuxi, Chongqing City | 109°34' | 31°23' | 1596 |
| HB-10 | *Fritillaria hupehensis* | Fritillariae hupehensis bulbus | Original Plant | Wuxi, Chongqing City | 109°34' | 31°23' | 1596 |
| PBM-01 | *Fritillaria ussuriensis* | Fritillariae ussuriensis bulbus | Original Plant | Yichun, Heilongjiang Province | 127°40' | 46°28' | 1228 |
| PBM-02 | *Fritillaria ussuriensis* | Fritillariae ussuriensis bulbus | Original Plant | Yichun, Heilongjiang Province | 127°40' | 46°28' | 1228 |
| PBM-03 | *Fritillaria ussuriensis* | Fritillariae ussuriensis bulbus | Original Plant | Yichun, Heilongjiang Province | 127°40' | 46°28' | 1228 |
| PBM-04 | *Fritillaria ussuriensis* | Fritillariae ussuriensis bulbus | Original Plant | Yichun, Heilongjiang Province | 127°40' | 46°28' | 1228 |
| PBM-05 | *Fritillaria ussuriensis* | Fritillariae ussuriensis bulbus | Original Plant | Yichun, Heilongjiang Province | 127°40' | 46°28' | 1228 |
| PBM-06 | *Fritillaria ussuriensis* | Fritillariae ussuriensis bulbus | Original Plant | Yichun, Heilongjiang Province | 127°40' | 46°28' | 1228 |
| PBM-07 | *Fritillaria ussuriensis* | Fritillariae ussuriensis bulbus | Original Plant | Yichun, Heilongjiang Province | 127°40' | 46°28' | 1228 |
| PBM-08 | *Fritillaria ussuriensis* | Fritillariae ussuriensis bulbus | Original Plant | Yichun, Heilongjiang Province | 127°40' | 46°28' | 1228 |
| PBM-09 | *Fritillaria ussuriensis* | Fritillariae ussuriensis bulbus | Original Plant | Yichun, Heilongjiang Province | 127°40' | 46°28' | 1228 |
| PBM-10 | *Fritillaria ussuriensis* | Fritillariae ussuriensis bulbus | Original Plant | Yichun, Heilongjiang Province | 127°40' | 46°28' | 1228 |
| YL-01 | *Fritillaria pallidiflora* | Fritillariae pallidiflorae bulbus | Original Plant | Yichun, Heilongjiang Province | 127°40' | 46°28' | 1228 |
| YL-02 | *Fritillaria pallidiflora* | Fritillariae pallidiflorae bulbus | Original Plant | Changji, Xinjiang Province | 85°34′ | 43°28′ | 2145 |
| YL-03 | *Fritillaria pallidiflora* | Fritillariae pallidiflorae bulbus | Original Plant | Changji, Xinjiang Province | 85°34′ | 43°28′ | 2145 |
| YL-04 | *Fritillaria pallidiflora* | Fritillariae pallidiflorae bulbus | Original Plant | Changji, Xinjiang Province | 85°34′ | 43°28′ | 2145 |
| YL-05 | *Fritillaria pallidiflora* | Fritillariae pallidiflorae bulbus | Original Plant | Changji, Xinjiang Province | 85°34′ | 43°28′ | 2145 |
| YL-06 | *Fritillaria pallidiflora* | Fritillariae pallidiflorae bulbus | Original Plant | Changji, Xinjiang Province | 85°34′ | 43°28′ | 2145 |
| YL-07 | *Fritillaria pallidiflora* | Fritillariae pallidiflorae bulbus | Original Plant | Changji, Xinjiang Province | 85°34′ | 43°28′ | 2145 |
| YL-08 | *Fritillaria pallidiflora* | Fritillariae pallidiflorae bulbus | Original Plant | Changji, Xinjiang Province | 85°34′ | 43°28′ | 2145 |
| YL-09 | *Fritillaria pallidiflora* | Fritillariae pallidiflorae bulbus | Original Plant | Changji, Xinjiang Province | 85°34′ | 43°28′ | 2145 |
| YL-10 | *Fritillaria pallidiflora* | Fritillariae pallidiflorae bulbus | Original Plant | Changji, Xinjiang Province | 85°34′ | 43°28′ | 2145 |
| XJ-01 | *Fritillaria walujewii* | Fritillariae pallidiflorae bulbus | Original Plant | Changji, Xinjiang Province | 88°30′ | 45°30′ | 1956 |
| XJ-02 | *Fritillaria walujewii* | Fritillariae pallidiflorae bulbus | Original Plant | Changji, Xinjiang Province | 88°30′ | 45°30′ | 1956 |
| XJ-03 | *Fritillaria walujewii* | Fritillariae pallidiflorae bulbus | Original Plant | Changji, Xinjiang Province | 88°30′ | 45°30′ | 1956 |
| XJ-04 | *Fritillaria walujewii* | Fritillariae pallidiflorae bulbus | Original Plant | Changji, Xinjiang Province | 88°30′ | 45°30′ | 1956 |
| XJ-05 | *Fritillaria walujewii* | Fritillariae pallidiflorae bulbus | Original Plant | Changji, Xinjiang Province | 88°30′ | 45°30′ | 1956 |
| XJ-06 | *Fritillaria walujewii* | Fritillariae pallidiflorae bulbus | Original Plant | Changji, Xinjiang Province | 88°30′ | 45°30′ | 1956 |
| XJ-07 | *Fritillaria walujewii* | Fritillariae pallidiflorae bulbus | Original Plant | Changji, Xinjiang Province | 88°30′ | 45°30′ | 1956 |
| XJ-08 | *Fritillaria walujewii* | Fritillariae pallidiflorae bulbus | Original Plant | Changji, Xinjiang Province | 88°30′ | 45°30′ | 1956 |
| XJ-09 | *Fritillaria walujewii* | Fritillariae pallidiflorae bulbus | Original Plant | Changji, Xinjiang Province | 88°30′ | 45°30′ | 1956 |
| XJ-10 | *Fritillaria walujewii* | Fritillariae pallidiflorae bulbus | Original Plant | Changji, Xinjiang Province | 88°30′ | 45°30′ | 1956 |
| ZPM-01 | *Fritillaria thunbergii* | Fritillariae thunbergii bulbus | Original Plant | Lishui, Zhejiang Province | 27°57' | 119°59' | 530 |
| ZPM-02 | *Fritillaria thunbergii* | Fritillariae thunbergii bulbus | Original Plant | Lishui, Zhejiang Province | 27°57' | 119°59' | 530 |
| ZPM-03 | *Fritillaria thunbergii* | Fritillariae thunbergii bulbus | Original Plant | Lishui, Zhejiang Province | 27°57' | 119°59' | 530 |
| ZPM-04 | *Fritillaria thunbergii* | Fritillariae thunbergii bulbus | Original Plant | Lishui, Zhejiang Province | 27°57' | 119°59' | 530 |
| ZPM-05 | *Fritillaria thunbergii* | Fritillariae thunbergii bulbus | Original Plant | Lishui, Zhejiang Province | 27°57' | 119°59' | 530 |
| ZPM-06 | *Fritillaria thunbergii* | Fritillariae thunbergii bulbus | Original Plant | Lishui, Zhejiang Province | 27°57' | 119°59' | 530 |
| ZPM-07 | *Fritillaria thunbergii* | Fritillariae thunbergii bulbus | Original Plant | Lishui, Zhejiang Province | 27°57' | 119°59' | 530 |
| ZPM-08 | *Fritillaria thunbergii* | Fritillariae thunbergii bulbus | Original Plant | Lishui, Zhejiang Province | 27°57' | 119°59' | 530 |
| ZPM-09 | *Fritillaria thunbergii* | Fritillariae thunbergii bulbus | Original Plant | Lishui, Zhejiang Province | 27°57' | 119°59' | 530 |
| ZPM-10 | *Fritillaria thunbergii* | Fritillariae thunbergii bulbus | Original Plant | Lishui, Zhejiang Province | 27°57' | 119°59' | 530 |

Supplementary Table 2 PCR and sequencing primers for pyrosequencing

| Group NO. | SNP sites | The use of primers | Primer name | Direction | Primer sequences (5′-3′) |
| --- | --- | --- | --- | --- | --- |
| 1 | ITS 341 | PCR | CBF1 | Forward | ATGGGCACGACGAGTGGT |
|  |  | PCR | CBR1 | Reverse | biotin-GGTCCGGGTCTCTTGAGC |
|  |  | Sequencing | CBS1 | Forward | GAGCACCAGCAGGAT |
| 2 | ITS 361/366 | PCR | PZF1 | Forward | ATGGGCACGACGAGTGGT |
|  |  | PCR | PZR1 | Reverse | biotin-GGCACGCCCTCCTCGTAC |
|  |  | Sequencing | PZS1 | Forward | TCGTGGCCCCCCGTC |
| 3 | *mat*K 923 | PCR | AHF1 | Forward | TCCAGAAGATGTTGATCGTAAATA |
|  |  | PCR | AHR1 | Reverse | biotin-TACATTTATTGCGATTCTTTCTAC |
|  |  | Sequencing | AHS1 | Forward | TTCGCATTCAGATACATAAG |
| 4 | *mat*K 1173 | PCR | XYF1 | Forward | ACCAAAATTTCAAAATGGATAGGA |
|  |  | PCR | XYR1 | Reverse | biotin-CAAAGGGTTTTTCAGTCATTGTG |
|  |  | Sequencing | XYS1 | Forward | ATCCTAAATTCTGAG |

Supplementary Table 3a Collection of ITS sequences of *Fritillaria*

| NO. | Genus | Species | Sequence number in NCBI | Self-test sequence number | Retrieved genbank ID and sample No. |
| --- | --- | --- | --- | --- | --- |
| 1 | *Fritillaria* | *F. acmopetala* | 1 | / | AY616709.1 |
| 2 | *Fritillaria* | *F. affinis* | 8 | / | AY616710.1, MW025082.1, MW025083.1, MW025084.1, MW025085.1, MW025086.1, MW025087.1, MW025088.1 |
| 3 | *Fritillaria* | *F. agrestis* | 2 | / | AY616711.1, MW025089.1 |
| 4 | *Fritillaria* | *F. alburyana* | 1 | / | AY616712.1 |
| 5 | *Fritillaria* | *F. anhuiensis* | 5 | / | KF906204.1, KF906205.1, KF906206.1, MF083532.1, MH588430.1 |
| 6 | *Fritillaria* | *F. atropurpurea* | 1 | / | MW025090.1 |
| 7 | *Fritillaria* | *F. aurea* | 1 | / | AY616713.1 |
| 8 | *Fritillaria* | *F. biflora* | 2 | / | MW025091.1, MW025092.1 |
| 9 | *Fritillaria* | *F. borealis* | 3 | / | MK630301.1, MK630305.1, MK630306.1 |
| 10 | *Fritillaria* | *F. brandegeei* | 1 | / | MW025093.1 |
| 11 | *Fritillaria* | *F. camtschatcensis* | 1 | / | AY616714.1 |
| 12 | *Fritillaria* | *F. camschatcensis* | 4 | / | MG215622.1, MG216030.1, MG216565.1, MW025094.1 |
| 13 | *Fritillaria* | *F. caucasica* | 1 | / | AY616715.1 |
| 14 | *Fritillaria* | *F. charybdae* | 1 | / | MK630290.1 |
| 15 | *Fritillaria* | *F. chitralensis* | 1 | / | AY616716.1 |
| 16 | *Fritillaria* | *F. cirrhosa* | 107 | 20 | FJ514499.1, GQ205113.1, GQ205117.1, GQ205118.1, GQ205120.1, GQ205121.1, GQ205122.1, HM045469.1, JF778850.1, KF906207.1, KP711996.1, KP711997.1, KP711998.1, KT861545.1, KT861546.1, KT861547.1, KT861548.1, KT861549.1, KT861550.1, KT008126.1, KT008127.1, KT008128.1, KT008129.1, KT008130.1, KT008131.1, KT008132.1, KT008133.1, KT008134.1, KT008135.1, KT008136.1, KT008137.1, KT008138.1, KT008139.1, KT008140.1, KT008141.1, KT008142.1, KT008143.1, KT008144.1, KT008145.1, KT008146.1, KT008147.1, KT008148.1, KT008149.1, MF096336.1, MF096337.1, MF096338.1, MF096339.1, MF096351.1, MF096352.1, MF083537.1, MF083538.1, MF083539.1, MF083541.1, MF083542.1, MF083543.1, MF083544.1, MF083545.1, MF083546.1, MG525329.1, MN121633.1, MN121694.1, MN121831.1, MN184744.1, MN184743.1, MN184746.1, MN184750.1, MN184754.1, MN184798.1, MN184797.1, MN184799.1, MN184796.1, MN184801.1, MN736438.1, MN860224.1, MH588404.1, MH588405.1, MH588406.1, MH588407.1, MH588408.1, MH588409.1, MW828318.1, MW832495.1, MW832496.1, MW832497.1, MZ491217.1, OP758186.1, OQ215385.1, OQ215386.1, OQ215387.1, OQ215388.1, OQ215389.1, OQ215390.1, OQ215391.1, OQ215392.1, OQ215393.1, OQ215394.1, OQ215395.1, OQ215396.1, OQ215397.1, OQ215398.1, OQ215399.1, OQ215400.1, OQ215401.1, OQ215402.1, OQ215403.1, OQ215404.1, MT539156.1, MN736441.1, JY-01, JY-02, JY-03, JY-04, JY-05, JY-06, JY-07, JY-08, JY-09, JY-10, JY-11, JY-12, JY-13, JY-14, JY-15, JY-16, JY-17, JY-18, JY-19, JY-20 |
| 17 | *Fritillaria* | *F. crassicaulis* | 2 | / | KF906208.1, MF083540.1 |
| 18 | *Fritillaria* | *F. crassifolia* | 11 | / | AY616717.1, KM435150.1, KM435178.1, KM435179.1, KM435180.1, KM435181.1, KM435182.1, KM435183.1, KM435184.1, KM435185.1, KM435186.1 |
| 19 | *Fritillaria* | *F. dagana* | 10 | / | MW025095.1, OQ244464.1, OQ244465.1, OQ244466.1, OQ244467.1, OQ244468.1, OQ244469.1, OQ244470.1, OQ244471.1, OQ244472.1 |
| 20 | *Fritillaria* | *F. dajinensis* | 2 | / | GQ205114.1, MF083547.1 |
| 21 | *Fritillaria* | *F. davidi* | 1 | / | AY616718.1 |
| 22 | *Fritillaria* | *F. davidii* | 4 | / | FJ514500.1, MF083554.1, MF083555.1, MF083556.1 |
| 23 | *Fritillaria* | *F. delavayi* | 36 | 20 | GQ205130.1, JF778853.1, KP711999.1, KT008199.1, KT008200.1, KT008201.1, KT008202.1, KT008203.1, KT030251.1, MF096330.1, MF096334.1, MF096335.1, MF096348.1, MF096349.1, MF096350.1, MF083557.1, MN153738.1, MN153740.1, MN153749.1, MN153752.1, MN153754.1, MN184745.1, MN184747.1, MN184751.1, MN184753.1, MN184756.1, MN184800.1, MN184803.1, MN184802.1, MN184804.1, MN184807.1, MN184806.1, MN184748.1, MN736445.1, MH588418.1, MH588419.1, MH588420.1, SS-01, SS-02, SS-03, SS-04, SS-05, SS-06, SS-07, SS-08, SS-09, SS-10, SS-11, SS-12, SS-13, SS-14, SS-15, SS-16, SS-17, SS-18, SS-19, SS-20 |
| 24 | *Fritillaria* | *F. eastwoodiae* | 2 | / | AY616719.1, MW025096.1 |
| 25 | *Fritillaria* | *F. ebeiensis* | 4 | / | KY884651.1, MH293594.1, MH293595.1, MH293596.1 |
| 26 | *Fritillaria* | *F. falcata* | 2 | / | AY616720.1, MW025097.1 |
| 27 | *Fritillaria* | *F. ferganensis* | 1 | / | KC801058.1 |
| 28 | *Fritillaria* | *F. formica* | 2 | / | MK630295.1, MK630289.1 |
| 29 | *Fritillaria* | *F. gentneri* | 2 | / | AY616721.1, MW025098.1, MW025099.1, MW025100.1 |
| 30 | *Fritillaria* | *F. gibbosa* | 3 | / | AY616722.1, KM435231.1, KM435232.1 |
| 31 | *Fritillaria* | *F. glauca* | 2 | / | AY616723.1, MW025101.1 |
| 32 | *Fritillaria* | *F. gracilis* | 1 | / | MK630299.1 |
| 33 | *Fritillaria* | *F. haplostoma* | 1 | / | MK630304.1 |
| 34 | *Fritillaria* | *F. hermontis* | 1 | / | AY616724.1 |
| 35 | *Fritillaria* | *F. hupehensis* | 24 | 10 | GQ205124.1, KF906199.1, KF906200.1, KF906201.1, KF906202.1, KF906203.1, KT008165.1, KT008166.1, KT008167.1, KT008168.1, KT008169.1, KT008170.1, KT008171.1, KT008172.1, MN153739.1, MN153742.1, MN153746.1, MN153748.1, MN184752.1, MN184755.1, MN184759.1, MN184805.1, MN184808.1, MN184809.1, HB-01, HB-02, HB-03, HB-04, HB-05, HB-06, HB-07, HB-08, HB-09, HB-10 |
| 36 | *Fritillaria* | *F. imperialis* | 17 | / | AY616725.1, KM435147.1, KM435156.1, KM435157.1, KM435158.1, KM435159.1, KM435160.1, KM435161.1, KM435162.1, KM435163.1, KM435164.1, KM435165.1, KM435166.1, KM435167.1, KM435168.1, KM435169.1, KM435170.1 |
| 37 | *Fritillaria* | *F. involucrata* | 1 | / | MT522876.1 |
| 38 | *Fritillaria* | *F. japonica* | 2 | / | AY616726.1, LC634105.1 |
| 39 | *Fritillaria* | *F. karelini* | 1 | / | AY616727.1 |
| 40 | *Fritillaria* | *F. karelinii* | 9 | / | MG946135.1, MG946136.1, MG946137.1, MG946138.1, MG946139.1, MG946140.1, OP925892.1, OP925893.1, OQ629677.1 |
| 41 | *Fritillaria* | *F. koidzumiana* | 1 | / | LC634107.1 |
| 42 | *Fritillaria* | *F. kotschyana* | 2 | / | KM435233.1, KM435234.1 |
| 43 | *Fritillaria* | *F. latifolia* | 1 | / | AM292420.1 |
| 44 | *Fritillaria* | *F. liliacea* | 3 | / | MW025102.1, MW025103.1, MW025104.1 |
| 45 | *Fritillaria* | *F. lusitanica* | 1 | / | AY616728.1 |
| 46 | *Fritillaria* | *F. maximoviczii* | 2 | / | AY616729.1, HM045471.1 |
| 47 | *Fritillaria* | *F. maximowiczii* | 6 | / | KP712000.1, KT934331.1, KT934333.1, MG525328.1, OQ244473.1, OQ244474.1 |
| 48 | *Fritillaria* | *F. meleagris* | 2 | / | AY616730.1, MF543714.1 |
| 49 | *Fritillaria* | *F. meleagroides* | 6 | / | KC801074.1, MG946141.1, MG946142.1, MG946143.1, MG946144.1, MG946145.1 |
| 50 | *Fritillaria* | *F. mellea* | 3 | / | GQ205125.1, GQ205126.1, MN736439.1 |
| 51 | *Fritillaria* | *F. messanensis* | 1 | / | MK630302.1 |
| 52 | *Fritillaria* | *F. michailovskyi* | 1 | / | AY616731.1 |
| 53 | *Fritillaria* | *F. micrantha* | 2 | / | AY616732.1, MW025105.1 |
| 54 | *Fritillaria* | *F. minuta* | 1 | / | AY616733.1 |
| 55 | *Fritillaria* | *F. monantha* | 5 | / | KF906195.1, KF906196.1, KF906197.1, KF906198.1, MF083559.1 |
| 56 | *Fritillaria* | *F. montana* | 2 | / | MF543715.1, MF543716.1 |
| 57 | *Fritillaria* | *F. ojaiensis* | 2 | / | MW025106.1, MW025107.1 |
| 58 | *Fritillaria* | *F. olivieri* | 1 | / | AY616734.1 |
| 59 | *Fritillaria* | *F. omeiensis* | 2 | / | OP758188.1, OP758189.1 |
| 60 | *Fritillaria* | *F. pallidiflora* | 40 | 10 | AY616735.1, HQ010405.1, KC801072.1, KC801073.1, KT008213.1, KT008214.1, KT008215.1, KT008216.1, KT008217.1, KT008218.1, KT008219.1, KT008220.1, KT008221.1, KT008222.1, KT008223.1, MF096353.1, MF096354.1, MF096355.1, MF096356.1, MF096357.1, MF083561.1, MG946146.1, MG946147.1, MG946148.1, MG946149.1, MN121628.1, MN184757.1, MN184758.1, MN184760.1, MN184761.1, MN184762.1, MN184810.1, MN184817.1, MN184815.1, MN184816.1, MN184819.1, MH588431.1, MH588432.1, MH588433.1, MT975262.1, YL-01, YL-02, YL-03, YL-04, YL-05, YL-06, YL-07, YL-08, YL-09, YL-10 |
| 61 | *Fritillaria* | *F. pellucida* | 1 | / | MK630300.1 |
| 62 | *Fritillaria* | *F. persica* | 9 | / | AY616736.1, KM435148.1, KM435171.1, KM435172.1, KM435173.1, KM435174.1, KM435175.1, KM435176.1, KM435177.1 |
| 63 | *Fritillaria* | *F. phaeanthera* | 1 | / | AY616737.1 |
| 64 | *Fritillaria* | *F. pinetorum* | 1 | / | MW025108.1 |
| 65 | *Fritillaria* | *F. pluriflora* | 1 | / | MW025109.1 |
| 66 | *Fritillaria* | *F. polaris* | 1 | / | MK630288.1 |
| 67 | *Fritillaria* | *F. przewalskii* | 43 | 20 | GQ205115.1, JF778852.1, KF906210.1, KT861533.1, KT861534.1, KT861535.1, KT861536.1, KT861537.1, KT861538.1, KT008159.1, KT008160.1, KT008161.1, KT008162.1, KT008163.1, KT008164.1, MF096329.1, MF096331.1, MF096332.1, MF096333.1, MF096346.1, MF096347.1, KY884652.1, MF083551.1, MF083552.1, MG946150.1, MN153747.1, MN153750.1, MN153768.1, MN184763.1, MN184764.1, MN184765.1, MN184767.1, MN184769.1, MN184818.1, MN184821.1, MN184822.1, MN184826.1, MN184820.1, MN736442.1, MH588410.1, MH588411.1, MH588412.1, MH588413.1, GS-01, GS-02, GS-03, GS-04, GS-05, GS-06, GS-07, GS-08, GS-09, GS-10, GS-11, GS-12, GS-13, GS-14, GS-15, GS-16, GS-17, GS-18, GS-19, GS-20 |
| 68 | *Fritillaria* | *F. pudica* | 3 | / | AY616738.1, MG216681.1, MW025110.1 |
| 69 | *Fritillaria* | *F. puqiensis* | 1 | / | KY884650.1 |
| 70 | *Fritillaria* | *F. purdyi* | 2 | / | MW025111.1, MW025112.1 |
| 71 | *Fritillaria* | *F. qimenensis* | 1 | / | KY884648.1 |
| 72 | *Fritillaria* | *F. raddeana* | 6 | / | AY616739.1, KM435149.1, KM435152.1, KM435153.1, KM435154.1, KM435155.1 |
| 73 | *Fritillaria* | *F. ragusina* | 1 | / | MK630291.1 |
| 74 | *Fritillaria* | *F. recurva* | 2 | / | AY616740.1, MW025113.1 |
| 75 | *Fritillaria* | *F. reuteri* | 3 | / | AY616741.1 |
| 76 | *Fritillaria* | *F. roderickii* | 1 | / | MW025117.1 |
| 77 | *Fritillaria* | *F. sewerzowii* | 1 | / | AY616742.1 |
| 78 | *Fritillaria* | *F. sichuanica* | 3 | / | KP712001.1, MF083553.1, OP758187.1 |
| 79 | *Fritillaria* | *F. sinica* | 2 | / | KF906211.1, MN736440.1 |
| 80 | *Fritillaria* | *F. sonnikovae* | 3 | / | OP558092.1, OQ244475.1, OQ244476.1 |
| 81 | *Fritillaria* | *F. sp.* | 6 | / | MK630294.1, MK630303.1, MK630293.1, MW025114.1, MW025115.1, MW025116.1 |
| 82 | *Fritillaria* | *F. stenanthera* | 1 | / | AF092514.1 |
| 83 | *Fritillaria* | *F. straussii* | 1 | / | KM435238.1 |
| 84 | *Fritillaria* | *F. striata* | 3 | / | AY616743.1, MW025118.1, MW025119.1 |
| 85 | *Fritillaria* | *F. sulcisquamosa* | 1 | / | GQ205128.1 |
| 86 | *Fritillaria* | *F. taipaiensis* | 17 | 20 | HM045470.1, KP712002.1, KT861551.1, KT861552.1, KT861553.1, KT008150.1, KT008151.1, MF083558.1, MH711398.1, MN184768.1, MN184824.1, MN736443.1, MH588421.1, MH588422.1, MH588423.1, MH588424.1, MH588425.1, TB-01, TB-02, TB-03, TB-04, TB-05, TB-06, TB-07, TB-08, TB-09, TB-10, TB-11, TB-12, TB-13, TB-14, TB-15, TB-16, TB-17, TB-18, TB-19, TB-20 |
| 87 | *Fritillaria* | *F. tenella* | 2 | / | AY616744.1, MK630298.1 |
| 88 | *Fritillaria* | *F. thunbergii* | 57 | 10 | AM777880.1, GQ205129.1, HQ448863.1, KM051453.2, KP712003.1, KT030227.1, KT030228.1, KT030229.1, KT030230.1, KT030231.1, KT030232.1, KT030233.1, KT030234.1, KT030235.1, KT030236.1, KT030237.1, KT030238.1, KT030239.1, KT030240.1, KT030241.1, KT030242.1, KT030243.1, KT030244.1, KT030245.1, KT030246.1, KT030247.1, KT030248.1, KT030249.1, KT030250.1, KX674829.1, KX674978.1, KX675100.1, MF096323.1, MF096324.1, MF096325.1, MF096326.1, MF096327.1, MF096328.1, KY884644.1, KY884645.1, KY884646.1, KY884647.1, MF083549.1, MN184766.1, MN184771.1, MN184773.1, MN184776.1, MN184825.1, MN184823.1, MN184827.1, MN184829.1, MN184828.1, MN184770.1, MH588427.1, MH588428.1, MH588429.1, MF083548.1, ZPM-01, ZPM-02, ZPM-03, ZPM-04, ZPM-05, ZPM-06, ZPM-07, ZPM-08, ZPM-09, ZPM-10 |
| 89 | *Fritillaria* | *F. tortifolia* | 5 | / | KP712004.1, MG946151.1, MG946152.1, MG946153.1, MG946154.1 |
| 90 | *Fritillaria* | *F. tubiformis* | 6 | / | AY616745.1, MT522878.1, MT522879.1, MT522875.1, MT522874.1, MT522877.1 |
| 91 | *Fritillaria* | *F. unibracteata* | 69 | 20 | GQ205123.1, GQ205127.1, HQ448866.1, JF778851.1, KP712005.1, KP712006.1, KT861539.1, KT861540.1, KT861541.1, KT861542.1, KT861543.1, KT861544.1, KT008152.1, KT008153.1, KT008154.1, KT008155.1, KT008156.1, KT008157.1, KT008158.1, MF096340.1, MF096341.1, MF096342.1, MF096343.1, MF096344.1, MF096345.1, MF083533.1, MF083534.1, MF083535.1, MF083536.1, MG946155.1, MN153751.1, MN153753.1, MN153763.1, MN153767.1, MN184775.1, MN184774.1, MN184777.1, MN184778.1, MN184781.1, MN184830.1, MN184837.1, MN184842.1, MN184835.1, MN184843.1, MN736437.1, MH588414.1, MH588415.1, MH588416.1, MH588417.1, OQ215458.1, OQ215459.1, OQ215460.1, OQ215461.1, OQ215462.1, OQ215463.1, OQ215464.1, OQ215465.1, OQ215466.1, OQ215467.1, OQ215468.1, OQ215469.1, OQ215470.1, OQ215471.1, OQ215472.1, OQ215473.1, OQ215474.1, OQ215475.1, OQ215476.1, OQ215477.1, AZ-01, AZ-02, AZ-03, AZ-04, AZ-05, AZ-06, AZ-07, AZ-08, AZ-09, AZ-10, AZ-11, AZ-12, AZ-13, AZ-14, AZ-15, AZ-16, AZ-17, AZ-18, AZ-19, AZ-20 |
| 92 | *Fritillaria* | *F. unibracteata var. longinectarea* | 6 | / | KT008204.1, KT008205.1, KT008206.1, KT008207.1, KT008208.1, KT008209.1, KT008210.1, KX669649.1, KX669650.1, MN736444.1 |
| 93 | *Fritillaria* | *F. unibracteata var. wabuensis* | 11 | 20 | GQ205116.1, GQ205119.1, KF906209.1, MG525330.1, MW809389.1, JF778854.1, WB-01, WB-02, WB-03, WB-04, WB-05, WB-06, WB-07, WB-08, WB-09, WB-10, WB-11, WB-12, WB-13, WB-14, WB-15, WB-16, WB-17, WB-18, WB-19, WB-20 |
| 94 | *Fritillaria* | *F. urticans* | 2 | / | MK630292.1 |
| 95 | *Fritillaria* | *F. ussuriensis* | 51 | 10 | DQ191622.1, JF778855.1, KT008173.1, KT008174.1, KT008175.1, KT008176.1, KT008177.1, KT008178.1, KT008179.1, KT008180.1, KT008181.1, KT008182.1, KT008183.1, KT008184.1, KT008185.1, KT008186.1, KT008187.1, KT008188.1, KT008189.1, KT008190.1, KT008191.1, KT008192.1, KT008193.1, KT008194.1, KT008195.1, KT008196.1, KT008197.1, KT008198.1, MG946156.1, MK726258.1, MK726259.1, MK726260.1, MK726261.1, MK726275.1, MN153741.1, MN153761.1, MN153764.1, MN153765.1, MN184772.1, MN184779.1, MN184780.1, MN184783.1, MN184784.1, MN184832.1, MN184833.1, MN184838.1, MN184840.1, MN184841.1, MH588434.1, MH588435.1, MH588436.1, PBM-01, PBM-02, PBM-03, PBM-04, PBM-05, PBM-06, PBM-07, PBM-08, PBM-09, PBM-10 |
| 96 | *Fritillaria* | *F. venusta* | 1 | / | MK630297.1 |
| 97 | *Fritillaria* | *F. verticillata* | 10 | / | KC801055.1, KC801056.1, KC801066.1, KC801067.1, KC801068.1, KC801069.1, KP712007.1, MF083550.1, MG946157.1, MG946158.1 |
| 98 | *Fritillaria* | *F. viridea* | 2 | / | MW025120.1, MW025121.1 |
| 99 | *Fritillaria* | *F. walujewii* | 19 | 10 | KC801070.1, KC801071.1, KP712008.1, KT008211.1, KT008212.1, MF083560.1, MG946159.1, MG946160.1, MG946161.1, MG946162.1, MG946163.1, MN184782.1, MN184786.1, MN184787.1, MN184788.1, MN184836.1, MN184839.1, MN184831.1, MN184834.1, XJ-01, XJ-02, XJ-03, XJ-04,XJ-05,XJ-06, XJ-07, XJ-08,XJ-09, XJ-10 |
| 100 | *Fritillaria* | *F. wanjiangensis* | 1 | / | KY884649.1 |
| 101 | *Fritillaria* | *F. yuminensis* | 9 | / | HM045472.1, KC801064.1, KC801065.1, MG946164.1, MG946165.1, MG946166.1, MG946167.1, MG946168.1, MG946169.1 |
| 102 | *Fritillaria* | *F. yuzhongensis* | 1 | / | MF083562.1 |
| 103 | *Fritillaria* | *F. zagrica* | 2 | / | KM435235.1, KM435236.1 |

Supplementary Table 3b Collection of *psb*A-*trn*H sequences of *Fritillaria*

| NO. | Genus | Species | Sequence number in NCBI | Self-test sequence number | Retrieved genbank ID and sample No. |
| --- | --- | --- | --- | --- | --- |
| 1 | *Fritillaria* | *F. affinis* | 1 | / | GQ248307.1 |
| 2 | *Fritillaria* | *F. anhuiensis* | 7 | / | MK258148.1, MH593363.1, MN810977.1, MN810978.1, MN810979.1, MN810980.1, MN810981.1 |
| 3 | *Fritillaria* | *F. ariana* | 1 | / | KU159159.1 |
| 4 | *Fritillaria* | *F. assyriaca* | 1 | / | KU159168.1 |
| 5 | *Fritillaria* | *F. atrolineata* | 1 | / | KU159173.1 |
| 6 | *Fritillaria* | *F. avromanica* | 1 | / | KU159169.1 |
| 7 | *Fritillaria* | *F. camschatcensis* | 1 | / | MW794313.1 |
| 8 | *Fritillaria* | *F. caucasica* | 1 | / | KU159171.1 |
| 9 | *Fritillaria* | *F. chlorantha* | 1 | / | KU159172.1 |
| 10 | *Fritillaria* | *F. cirrhosa* | 37 | / | HQ662561.1, KF850863.1, KF769143.1, KY646167.1, MH244906.1, MN721887.1, MH593342.1, MH593343.1, MH593344.1, MH593345.1, MH593346.1, MT806755.1, OK081990.1, OK081991.1, OK081992.1, OK081993.1, MZ540188.1, OQ147426.1, OQ147427.1, OQ147428.1, OQ147429.1, OQ147430.1, OQ147431.1, OQ147432.1, OQ147433.1, OQ147434.1, OQ147435.1, OQ147436.1, OQ147437.1, OQ147438.1, OQ147439.1, OQ147440.1, OQ147441.1, OQ147442.1, OQ147443.1, OQ147444.1, OQ147445.1 |
| 11 | *Fritillaria* | *F. cirrhosa var. ecirrhosa* | 1 | / | MN721890.1 |
| 12 | *Fritillaria* | *F. crassicaulis* | 4 | / | MK258147.1, MN810998.1, MN810999.1, MN811000.1 |
| 13 | *Fritillaria* | *F. crassifolia* | 3 | / | KU159165.1, KU159166.1, KU159167.1 |
| 14 | *Fritillaria* | *F. crassifolia subsp. poluninii* | 1 | / | KU159175.1 |
| 15 | *Fritillaria* | *F. dagana* | 5 | / | OQ267647.1, OQ267648.1, OQ267649.1, OQ267650.1, OQ267651.1 |
| 16 | *Fritillaria* | *F. dajinensis* | 4 | / | MH244913.1, MK258146.1, MN810972.1, MN810973.1 |
| 17 | *Fritillaria* | *F. davidii* | 4 | / | MK258145.1, MN810982.1, MN810983.1, MN810984.1 |
| 18 | *Fritillaria* | *F. delavayi* | 6 | / | MK258144.1, MN721894.1, MH593353.1, MH593354.1, MH593355.1, MN480806.1 |
| 19 | *Fritillaria* | *F. eduardii* | 1 | / | MF947708.1 |
| 20 | *Fritillaria* | *F. fusca* | 1 | / | MW890003.1 |
| 21 | *Fritillaria* | *F. gibbosa* | 1 | / | KU159158.1 |
| 22 | *Fritillaria* | *F. hupehensis* | 4 | / | KF712486.1, MN811001.1, MN811002.1, MN811003.1 |
| 23 | *Fritillaria* | *F. imperialis* | 2 | / | KU159155.1, KU159156.1 |
| 24 | *Fritillaria* | *F. karelinii* | 7 | / | KX354691.1, MG211818.1, MG211821.1, MZ970254.1, OP938257.1, OP938258.1, OP938259.1 |
| 25 | *Fritillaria* | *F. maximowiczii* | 6 | / | MK258138.1, MN810992.1, MN810993.1, MN810994.1, OQ267652.1, OQ267653.1 |
| 26 | *Fritillaria* | *F. meleagris* | 1 | / | MF543650.1 |
| 27 | *Fritillaria* | *F. meleagroides* | 1 | / | MF947710.1 |
| 28 | *Fritillaria* | *F. mellea* | 1 | / | MN721888.1 |
| 29 | *Fritillaria* | *F. micrantha* | 1 | / | GQ248308.1 |
| 30 | *Fritillaria* | *F. monantha* | 4 | / | MK258143.1, MN810974.1, MN810975.1, MN810976.1 |
| 31 | *Fritillaria* | *F. montana* | 5 | / | MF543651.1, MF543652.1, MF543653.1, MF543654.1, MZ540183.1 |
| 32 | *Fritillaria* | *F. olivieri* | 1 | / | KU159162.1 |
| 33 | *Fritillaria* | *F. omeiensis* | 3 | / | MN810995.1, MN810996.1, MN810997.1 |
| 34 | *Fritillaria* | *F. pallidiflora* | 4 | / | KJ956421.1, MG211822.1, MH593364.1, MH593365.1, MH593366.1 |
| 35 | *Fritillaria* | *F. persica* | 4 | / | EU939294.1, KU159160.1, KU159161.1, MF947709.1 |
| 36 | *Fritillaria* | *F. pinardii* | 1 | / | KU159176.1 |
| 37 | *Fritillaria* | *F. przewalskii* | 7 | / | MH244908.1, MK258142.1, MN721891.1, MH593347.1, MH593348.1, MH593349.1, MT806747.1 |
| 38 | *Fritillaria* | *F. raddeana* | 1 | / | KU159157.1 |
| 39 | *Fritillaria* | *F. reuteri* | 1 | / | KU159163.1 |
| 40 | *Fritillaria* | *F. sichuanica* | 5 | / | MH244907.1, MK258141.1, MT806748.1, MN810967.1, MN810968.1 |
| 41 | *Fritillaria* | *F. sinica* | 3 | / | MH244912.1, MN721889.1, MN810971.1 |
| 42 | *Fritillaria* | *F. sonnikovae* | 2 | / | OP672186.1, OQ267654.1 |
| 43 | *Fritillaria* | *F. straussii* | 1 | / | KU159164.1 |
| 44 | *Fritillaria* | *F. taipaiensis* | 20 | / | KC543997.1, KC713822.1, KC713823.1, KF769144.1, MH244910.1, MN721892.1, MH593356.1, MH593357.1, MH593358.1, MH593359.1, MK642356.1, MT806749.1, MT806750.1, MT806751.1, MT806752.1, MT806753.1, MT806754.1, OQ411258.1, OQ433930.1, OQ433931.1 |
| 45 | *Fritillaria* | *F. thunbergii* | 6 | / | KF850862.1, KY646165.1, MH244914.1, MH593360.1, MH593361.1, MH593362.1 |
| 46 | *Fritillaria* | *F. tortifolia* | 4 | / | MG211819.1, MN810985.1, MN810986.1, MN810987.1 |
| 47 | *Fritillaria* | *F. unibracteata* | 30 | / | GQ434876.1, MF096902.1, MH244909.1, MK258140.1, MN721886.1, MN727396.1, MH593350.1, MH593351.1, MH593352.1, OM807063.1, OQ147446.1, OQ147447.1, OQ147448.1, OQ147449.1, OQ147450.1, OQ147451.1, OQ147452.1, OQ147453.1, OQ147454.1, OQ147455.1, OQ147456.1, OQ147457.1, OQ147458.1, OQ147459.1, OQ147460.1, OQ147461.1, OQ147462.1, OQ147463.1, OQ147464.1, OQ147465.1 |
| 48 | *Fritillaria* | *F. unibracteata var. longinectarea* | 2 | / | OM807060.1, OM807061.1 |
| 49 | *Fritillaria* | *F. unibracteata var. maculata* | 1 | / | OM807062.1 |
| 50 | *Fritillaria* | *F. unibracteata var. wabuensis* | 2 | / | KF769142.1, MN721893.1 |
| 51 | *Fritillaria* | *F. ussuriensis* | 5 | / | KY646166.1, MH593367.1, MH593368.1, MH593369.1, MT261156.1 |
| 52 | *Fritillaria* | *F. uva-vulpis* | 1 | / | KU159170.1 |
| 53 | *Fritillaria* | *F. verticillata* | 1 | / | MG211823.1 |
| 54 | *Fritillaria* | *F. walujewii* | 6 | / | KJ956409.1, MG211820.1, MN810988.1, MN810989.1, MN810990.1, MN810991.1 |
| 55 | *Fritillaria* | *F. yuminensis* | 1 | / | MG200070.1 |
| 56 | *Fritillaria* | *F. yuzhongensis* | 4 | / | MH244911.1, MK258139.1, MN810969.1, MN810970.1 |
| 57 | *Fritillaria* | *F. zagrica* | 1 | / | KU159174.1 |

Supplementary Table 3c Collection of *rbc*L sequences of *Fritillaria*

| NO. | Genus | Species | Sequence number in NCBI | Self-test sequence number | Retrieved genbank ID and sample No. |
| --- | --- | --- | --- | --- | --- |
| 1 | *Fritillaria* | *F. acmopetala* | 2 | / | JN417429.1, KM085472.1 |
| 2 | *Fritillaria* | *F. acmopetala subsp. acmopetala* | 1 | / | LM992907.1 |
| 3 | *Fritillaria* | *F. affinis* | 8 | / | GQ248610.1, LM992908.1, LM992909.1, KM085473.1, KX677845.1, KX678163.1, KX678576.1, KX679156.1 |
| 4 | *Fritillaria* | *F. agrestis* | 2 | / | AF013233.1, LM992910.1 |
| 5 | *Fritillaria* | *F. alburyana* | 2 | / | LM992911.1, KM085474.1 |
| 6 | *Fritillaria* | *F. alfredae subsp. glaucoviridis* | 1 | / | LM992912.1 |
| 7 | *Fritillaria* | *F. amabilis* | 2 | / | LM992913.1, LM992914.1 |
| 8 | *Fritillaria* | *F. amana* | 2 | / | LM992915.1, LM992916.1 |
| 9 | *Fritillaria* | *F. anhuiensis* | 10 | / | KP768974.1, KP768975.1, MG525358.1, MK258148.1, MH593363.1, MN810977.1, MN810978.1, MN810979.1, MN810980.1, MN810981.1, |
| 10 | *Fritillaria* | *F. armena* | 1 | / | LM992917.1 |
| 11 | *Fritillaria* | *F. assyriaca subsp. assyriaca* | 2 | / | LM992918.1, LM992919.1 |
| 12 | *Fritillaria* | *F. atropurpurea* | 1 | / | LM992920.1 |
| 13 | *Fritillaria* | *F. aurea* | 1 | / | LM992921.1 |
| 14 | *Fritillaria* | *F. ayakoana* | 1 | / | LM992922.1 |
| 15 | *Fritillaria* | *F. bithynica* | 1 | / | LM992923.1 |
| 16 | *Fritillaria* | *F. bucharica* | 1 | / | LM992924.1 |
| 17 | *Fritillaria* | *F. camschatcensis* | 11 | / | LM992925.1, LM992926.1, LM992927.1, LM992928.1, LM992929.1, KM085475.1, MG225616.1, MG226537.1, MG227399.1, MG227744.1, MW794313.1 |
| 18 | *Fritillaria* | *F. carica* | 1 | / | LM992930.1 |
| 19 | *Fritillaria* | *F. caucasica* | 2 | / | EU606205.1, LM992931.1 |
| 20 | *Fritillaria* | *F. chitralensis* | 3 | / | JN417430.1, LM992932.1, KM085480.1 |
| 21 | *Fritillaria* | *F. cirrhosa* | 44 | / | KF850895.1, KF769143.1, LM992933.1, KM085476.1, KP711913.1, KP711914.1, KP711915.1, KT749070.1, KY646167.1, MG525360.1, MG525361.1, MH244906.1, MH593342.1, MH593343.1, MH593344.1, MH593345.1, MH593346.1, MT354770.1, MT806755.1, OK081985.1, OK081986.1, OK081987.1, OK081988.1, MZ540202.1, OQ117288.1, OQ117289.1, OQ117290.1, OQ117291.1, OQ117292.1, OQ117293.1, OQ117294.1, OQ117295.1, OQ117296.1, OQ117297.1, OQ117298.1, OQ117299.1, OQ117300.1, OQ117301.1, OQ117302.1, OQ117303.1, OQ117304.1, OQ117305.1, OQ117306.1, OQ117307.1, JY-01, JY-02, JY-03, JY-04, JY-05, JY-06, JY-07, JY-08, JY-09, JY-10, JY-11, JY-12, JY-13, JY-14, JY-15, JY-16, JY-17, JY-18, JY-19, JY-20 |
| 22 | *Fritillaria* | *F. conica* | 1 | / | LM992934.1 |
| 23 | *Fritillaria* | *F. crassicaulis* | 5 | / | MG525365.1, MK258147.1, MN810998.1, MN810999.1, MN811000.1 |
| 24 | *Fritillaria* | *F. crassifolia subsp. crassifolia* | 1 | / | LM992935.1 |
| 25 | *Fritillaria* | *F. crassifolia subsp. poluninii* | 1 | / | LM992988.1 |
| 26 | *Fritillaria* | *F. dagana* | 1 | / | LM992938.1 |
| 27 | *Fritillaria* | *F. dajinensis* | 5 | / | MG525368.1, MH244913.1, MK258146.1, MN810972.1, MN810973.1 |
| 28 | *Fritillaria* | *F. davidii* | 8 | / | LM992939.1, KM085477.1, KP998201.1, MG525352.1, MK258145.1, MN810982.1, MN810983.1, MN810984.1 |
| 29 | *Fritillaria* | *F. davisii* | 1 | / | LM992940.1 |
| 30 | *Fritillaria* | *F. delavayi* | 7 | / | KP711916.1, MG525366.1, MK258144.1, MH593353.1, MH593354.1, MH593355.1, MN480806.1, SS-01, SS-02, SS-03, SS-04, SS-05, SS-06, SS-07, SS-08, SS-09, SS-10, SS-11, SS-12, SS-13, SS-14, SS-15, SS-16, SS-17, SS-18, SS-19, SS-20 |
| 31 | *Fritillaria* | *F. drenovskii* | 1 | / | LM992941.1 |
| 32 | *Fritillaria* | *F. eastwoodiae* | 2 | / | LM992943.1, MF947708.1 |
| 33 | *Fritillaria* | *F. eduardii* | 2 | / | LM992943.1, MF947708.1 |
| 34 | *Fritillaria* | *F. ehrhartii* | 1 | / | LM992944.1 |
| 35 | *Fritillaria* | *F. elwesii* | 1 | / | LM992945.1 |
| 36 | *Fritillaria* | *F. falcata* | 1 | / | LM992946.1 |
| 37 | *Fritillaria* | *F. fleischeriana* | 1 | / | LM992947.1 |
| 38 | *Fritillaria* | *F. forbesii* | 1 | / | LM992948.1 |
| 39 | *Fritillaria* | *F. frankiorum* | 1 | / | LM992949.1 |
| 40 | *Fritillaria* | *F. fusca* | 1 | / | MW890003.1 |
| 41 | *Fritillaria* | *F. gibbosa* | 2 | / | LM992950.1, KM085479.1 |
| 42 | *Fritillaria* | *F. graeca* | 1 | / | LM992951.1 |
| 43 | *Fritillaria* | *F. gussichiae* | 1 | / | LM992952.1 |
| 44 | *Fritillaria* | *F. hupehensis* | 4 | / | KF712486.1, MN811001.1, MN811002.1, MN811003.1, HB-01, HB-02, HB-03, HB-04, HB-05, HB-06, HB-07, HB-08, HB-09, HB-10 |
| 45 | *Fritillaria* | *F. imperialis* | 3 | / | LM992953.1, KP998202.1, KY400627.1 |
| 46 | *Fritillaria* | *F. involucrata* | 2 | / | LM992954.1, LM992955.1 |
| 47 | *Fritillaria* | *F. japonica* | 3 | / | LM992956.1, KM085481.1, KP998203.1 |
| 48 | *Fritillaria* | *F. kaiensis* | 1 | / | LM992957.1 |
| 49 | *Fritillaria* | *F. karelinii* | 7 | / | KX354691.1, MG211818.1, MG211821.1, MZ969879.1, OP894661.1, OP894662.1, OP894663.1 |
| 50 | *Fritillaria* | *F. koidzumiana* | 4 | / | AB024390.1, AB034939.1, LM992958.1, KP998204.1 |
| 51 | *Fritillaria* | *F. kotschyana* | 2 | / | JN417431.1, LM992959.1 |
| 52 | *Fritillaria* | *F. kurdica* | 2 | / | LM992936.1, LM992937.1 |
| 53 | *Fritillaria* | *F. latakiensis* | 1 | / | LM992960.1 |
| 54 | *Fritillaria* | *F. latifolia* | 1 | / | LM992961.1 |
| 55 | *Fritillaria* | *F. liliacea* | 1 | / | LM992962.1 |
| 56 | *Fritillaria* | *F. lusitanica* | 1 | / | LM992963.1 |
| 57 | *Fritillaria* | *F. maximowiczii* | 8 | / | LM992964.1, LM992965.1, KP711917.1, MG525351.1, MK258138.1, MN810992.1, MN810993.1, MN810994.1 |
| 58 | *Fritillaria* | *F. meleagris* | 4 | / | AF276003.1, AY395537.1, KM360791.1, MF572199.1 |
| 59 | *Fritillaria* | *F. meleagris subsp. meleagris* | 1 | / | LM992966.1 |
| 60 | *Fritillaria* | *F. meleagroides* | 2 | / | LM992967.1, MF947710.1 |
| 61 | *Fritillaria* | *F. messanensis subsp. messanensis* | 1 | / | LM992968.1 |
| 62 | *Fritillaria* | *F. michailovskyi* | 1 | / | LM992969.1 |
| 63 | *Fritillaria* | *F. micrantha* | 3 | / | GQ248611.1, LM992970.1, KM085482.1 |
| 64 | *Fritillaria* | *F. minuta* | 1 | / | LM992971.1 |
| 65 | *Fritillaria* | *F. monantha* | 5 | / | MG525357.1, MK258143.1, MN810974.1, MN810975.1, MN810976.1 |
| 66 | *Fritillaria* | *F. montana* | 6 | / | LM992972.1, LM992973.1, MF572200.1, MF572201.1, MF572202.1, MF572203.1 |
| 67 | *Fritillaria* | *F. muraiana* | 2 | / | LM992974.1, LM992975.1 |
| 68 | *Fritillaria* | *F. mutabilis* | 1 | / | LM992976.1 |
| 69 | *Fritillaria* | *F. obliqua subsp. obliqua* | 1 | / | LM992977.1 |
| 70 | *Fritillaria* | *F. obliqua subsp. tuntasia* | 2 | / | LM992978.1, LM992979.1 |
| 71 | *Fritillaria* | *F. olivieri* | 1 | / | LM992980.1 |
| 72 | *Fritillaria* | *F. omeiensis* | 3 | / | MN810995.1, MN810996.1, MN810997.1 |
| 73 | *Fritillaria* | *F. oranensis* | 1 | / | LM992981.1 |
| 74 | *Fritillaria* | *F. orientalis* | 2 | / | LM992982.1, LM992983.1 |
| 75 | *Fritillaria* | *F. pallidiflora* | 5 | / | LM992984.1, MG211822.1, MH593364.1, MH593365.1, MH593366.1, YL-01, YL-02, YL-03, YL-04, YL-05, YL-06, YL-07, YL-08, YL-09, YL-10 |
| 76 | *Fritillaria* | *F. persica* | 3 | / | LM992985.1, KM085483.1, MF947709.1 |
| 77 | *Fritillaria* | *F. pinardii* | 1 | / | LM992986.1 |
| 78 | *Fritillaria* | *F. pluriflora* | 1 | / | LM992987.1 |
| 79 | *Fritillaria* | *F. pontica* | 1 | / | LM992989.1 |
| 80 | *Fritillaria* | *F. przewalskii* | 7 | / | MG525362.1, MH244908.1, MK258142.1, MH593347.1, MH593348.1, MH593349.1, MT806747.1, GS-01, GS-02, GS-03, GS-04, GS-05, GS-06, GS-07, GS-08, GS-09, GS-10, GS-11, GS-12, GS-13, GS-14, GS-15, GS-16, GS-17, GS-18, GS-19, GS-20 |
| 81 | *Fritillaria* | *F. pudica* | 5 | / | LM992990.1, KM085484.1, KX678295.1, KX679082.1, KX679178.1 |
| 82 | *Fritillaria* | *F. pyrenaica subsp. pyrenaica* | 1 | / | LM992991.1 |
| 83 | *Fritillaria* | *F. raddeana* | 2 | / | Z77293.1, LM992992.1 |
| 84 | *Fritillaria* | *F. recurva* | 1 | / | LM992993.1 |
| 85 | *Fritillaria* | *F. reuteri* | 1 | / | LM992994.1 |
| 86 | *Fritillaria* | *F. rixii* | 1 | / | LM992995.1 |
| 87 | *Fritillaria* | *F. ruthenica* | 1 | / | LM992996.1 |
| 88 | *Fritillaria* | *F. sewerzowii* | 4 | / | LM992997.1, LM992998.1, LM992999.1, KM085485.1 |
| 89 | *Fritillaria* | *F. shikokiana* | 2 | / | LM993000.1, LM993001.1 |
| 90 | *Fritillaria* | *F. sibthorpiana subsp. enginiana* | 1 | / | LM993002.1 |
| 91 | *Fritillaria* | *F. sichuanica* | 7 | / | KP711918.1, MG525367.1, MH244907.1, MK258141.1, MT806748.1, MN810967.1, MN810968.1 |
| 92 | *Fritillaria* | *F. sinica* | 2 | / | MH244912.1, MN810971.1 |
| 93 | *Fritillaria* | *F. sororum* | 2 | / | LM993003.1, LM993004.1 |
| 94 | *Fritillaria* | *F. stenanthera* | 1 | / | LM993005.1 |
| 95 | *Fritillaria* | *F. stribrnyi* | 1 | / | LM993006.1 |
| 96 | *Fritillaria* | *F. taipaiensis* | 22 | / | KC543997.1, KC713822.1, KC713823.1, KF769144.1, KP711919.1, MG525364.1, MH658417.1, MH244910.1, MH593356.1, MH593357.1, MH593358.1, MH593359.1, MK642356.1, MT806749.1, MT806750.1, MT806751.1, MT806752.1, MT806753.1, MT806754.1, OQ411258.1, OQ433930.1, OQ433931.1, TB-01, TB-02, TB-03, TB-04, TB-05, TB-06, TB-07, TB-08, TB-09, TB-10, TB-11, TB-12, TB-13, TB-14, TB-15, TB-16, TB-17, TB-18, TB-19, TB-20 |
| 97 | *Fritillaria* | *F. theophrasti* | 1 | / | LM993007.1 |
| 98 | *Fritillaria* | *F. thessala subsp. reiseri* | 1 | / | LM993008.1 |
| 99 | *Fritillaria* | *F. thessala subsp. thessala* | 1 | / | LM993009.1 |
| 100 | *Fritillaria* | *F. thunbergii* | 10 | / | KF850894.1, LM993010.1, KP711920.1, KY646165.1, MG525356.1, MH244914.1, MN431190.1, MH593360.1, MH593361.1, MH593362.1, ZPM-01, ZPM-02, ZPM-03, ZPM-04, ZPM-05, ZPM-06, ZPM-07, ZPM-08, ZPM-09, ZPM-10 |
| 101 | *Fritillaria* | *F. thunbergii var. chekiangensis* | 1 | / | MG525359.1 |
| 102 | *Fritillaria* | *F. tokushimensis* | 1 | / | LM993011.1 |
| 103 | *Fritillaria* | *F. tortifolia* | 7 | / | LM993012.1, KP711921.1, MG211819.1, MG525354.1, MN810985.1, MN810986.1, MN810987.1 |
| 104 | *Fritillaria* | *F. tubiformis subsp. tubiformis* | 1 | / | LM993013.1 |
| 105 | *Fritillaria* | *F. unibracteata* | 30 | / | KP711922.1, KP711923.1, MG525369.1, MH244909.1, MK258140.1, MH593350.1, MH593351.1, MH593352.1, OM807063.1, OQ117308.1, OQ117309.1, OQ117310.1, OQ117311.1, OQ117312.1, OQ117313.1, OQ117314.1, OQ117315.1, OQ117316.1, OQ117317.1, OQ117318.1, OQ117319.1, OQ117320.1, OQ117321.1, OQ117322.1, OQ117323.1, OQ117324.1, OQ117325.1, OQ117326.1, OQ117327.1, AZ-01, AZ-02, AZ-03, AZ-04, AZ-05, AZ-06, AZ-07, AZ-08, AZ-09, AZ-10, AZ-11, AZ-12, AZ-13, AZ-14, AZ-15, AZ-16, AZ-17, AZ-18, AZ-19, AZ-20 |
| 106 | *Fritillaria* | *F. unibracteat avar. longinectarea* | 3 | / | MG525370.1, OM807060.1, OM807061.1 |
| 107 | *Fritillaria* | *F. unibracteata var. maculata* | 1 | / | OM807062.1 |
| 108 | *Fritillaria* | *F. unibracteata var. wabuensis* | 1 | / | KF769142.1, WB-01, WB-02, WB-03, WB-04, WB-05, WB-06, WB-07, WB-08, WB-09, WB-10, WB-11, WB-12, WB-13, WB-14, WB-15, WB-16, WB-17, WB-18, WB-19, WB-20 |
| 109 | *Fritillaria* | *F. ussuriensis* | 9 | / | JN417432.1, LM993014.1, LM993015.1, KM085486.1, KY646166.1, MH593367.1, MH593368.1, MH593369.1, MT261156.1, PBM-01, PBM-02, PBM-03, PBM-04, PBM-05, PBM-06, PBM-07, PBM-08, PBM-09, PBM-10 |
| 110 | *Fritillaria* | *F. uva-vulpis* | 1 | / | LM993016.1 |
| 111 | *Fritillaria* | *F. verticillata* | 4 | / | LM993017.1, KP711924.1, MG211823.1, MG525355.1 |
| 112 | *Fritillaria* | *F. walujewii* | 8 | / | LM993018.1, KP711925.1, MG211820.1, MG525353.1, MN810988.1, MN810989.1, MN810990.1, MN810991.1, XJ-01, XJ-02, XJ-03, XJ-04,XJ-05,XJ-06, XJ-07, XJ-08,XJ-09, XJ-10 |
| 113 | *Fritillaria* | *F. whittallii* | 1 | / | LM993019.1 |
| 114 | *Fritillaria* | *F. yuminensis* | 1 | / | MG200070.1 |
| 115 | *Fritillaria* | *F. yuzhongensis* | 5 | / | MG525363.1, MH244911.1, MK258139.1, MN810969.1, MN810970.1 |
| 116 | *Fritillaria* | *F. zagrica* | 1 | / | LM993020.1 |

Supplementary Table 3d Collection of *mat*K sequences of *Fritillaria*

| NO. | Genus | Species | Sequence number in NCBI | Self-test sequence number | Retrieved genbank ID and sample No. |
| --- | --- | --- | --- | --- | --- |
| 1 | *Fritillaria* | *F. acmopetala* | 3 | / | AY624426.1, JN417340.1, KM085599.1 |
| 2 | *Fritillaria* | *F. affinis* | 6 | / | AY624427.1, LM993042.1, KM085600.1, KX676554.1, KX677181.1, KX677683.1 |
| 3 | *Fritillaria* | *F. agrestis* | 2 | / | AY624428.1, LM993043.1 |
| 4 | *Fritillaria* | *F. alburyana* | 2 | / | AY624429.1, KM085601.1 |
| 5 | *Fritillaria* | *F. alfredae subsp. glaucoviridis* | 1 | / | LM993044.1 |
| 6 | *Fritillaria* | *F. amabilis* | 2 | / | LM993045.1, LM993046.1 |
| 7 | *Fritillaria* | *F. amana* | 1 | / | LM993047.1 |
| 8 | *Fritillaria* | *F. anhuiensis* | 13 | / | KF906221.1, KF906222.1, KF906223.1, KP769182.1, KP769183.1, MG525338.1, MK258148.1, MH593363.1, MN810977.1, MN810978.1, MN810979.1, MN810980.1, MN810981.1 |
| 9 | *Fritillaria* | *F. armena* | 1 | / | LM993048.1 |
| 10 | *Fritillaria* | *F. assyriaca subsp. assyriaca* | 2 | / | LM993049.1, LM993050.1 |
| 11 | *Fritillaria* | *F. atropurpurea* | 1 | / | LM993051.1 |
| 12 | *Fritillaria* | *F. aurea* | 1 | / | AY624430.1 |
| 13 | *Fritillaria* | *F. ayakoana* | 1 | / | LM993052.1 |
| 14 | *Fritillaria* | *F. biflora* | 1 | / | MW081399.1 |
| 15 | *Fritillaria* | *F. bithynica* | 1 | / | LM993053.1 |
| 16 | *Fritillaria* | *F. bucharica* | 1 | / | LM993054.1 |
| 17 | *Fritillaria* | *F. camschatcensis* | 6 | / | AY624431.1, LM993055.1, LM993056.1, LM993057.1, LM993058.1, MW794313.1 |
| 18 | *Fritillaria* | *F. carica* | 1 | / | LM993059.1 |
| 19 | *Fritillaria* | *F. caucasica* | 1 | / | AY624432.1 |
| 20 | *Fritillaria* | *F. chitralensis* | 3 | / | AY624433.1, JN417341.1, KM085606.1 |
| 21 | *Fritillaria* | *F. cirrhosa* | 27 | 20 | GQ205132.1, GQ205136.1, GQ205137.1, GQ205139.1, GQ205140.1, GQ205141.1, JQ724625.1, KF850818.1, KF769143.1, KF906224.1, LM993060.1, KM085602.1, KP711862.1, KP711863.1, KP711864.1, KT749128.1, KY646167.1, MG525340.1, MG525341.1, MH244906.1, MH593342.1, MH593343.1, MH593344.1, MH593345.1, MH593346.1, MT806755.1, MZ556838.1 |
| 22 | *Fritillaria* | *F. conica* | 1 | / | LM993061.1 |
| 23 | *Fritillaria* | *F. crassicaulis* | 6 | / | KF906228.1, MG525345.1, MK258147.1, MN810998.1, MN810999.1, MN811000.1 |
| 24 | *Fritillaria* | *F. crassifolia* | 1 | / | AY624434.1 |
| 25 | *Fritillaria* | *F. crassifolia subsp. crassifolia* | 1 | / | LM993062.1 |
| 26 | *Fritillaria* | *F. crassifolia subsp. poluninii* | 1 | / | LM993105.1 |
| 27 | *Fritillaria* | *F. dagana* | 6 | / | LM993064.1, OQ267631.1, OQ267632.1, OQ267633.1, OQ267634.1, OQ267635.1 |
| 28 | *Fritillaria* | *F. dajinensis* | 6 | / | GQ205133.1, MG525348.1, MH244913.1, MK258146.1, MN810972.1, MN810973.1 |
| 29 | *Fritillaria* | *F. davidii* | 9 | / | AY624435.1, LM993065.1, KM085603.1, KP998197.1, MG525332.1, MK258145.1, MN810982.1, MN810983.1, MN810984.1, |
| 30 | *Fritillaria* | *F. davisii* | 1 | / | LM993066.1 |
| 31 | *Fritillaria* | *F. delavayi* | 8 | 20 | GQ205148.1, KP711865.1, MG525346.1, MK258144.1, MH593353.1, MH593354.1, MH593355.1, MN480806.1 |
| 32 | *Fritillaria* | *F. drenovskii* | 1 | / | LM993067.1 |
| 33 | *Fritillaria* | *F. eastwoodiae* | 2 | / | LM993068.1, KM085604.1 |
| 34 | *Fritillaria* | *F. ebeiensis* | 4 | / | KY884660.1, MH293597.1, MH293598.1, MH293599.1 |
| 35 | *Fritillaria* | *F. eduardii* | 2 | / | LM993069.1, MF947708.1 |
| 36 | *Fritillaria* | *F. ehrhartii* | 1 | / | LM993070.1 |
| 37 | *Fritillaria* | *F. elwesii* | 1 | / | LM993071.1 |
| 38 | *Fritillaria* | *F. falcata* | 1 | / | AY624436.1 |
| 39 | *Fritillaria* | *F. fleischeriana* | 1 | / | LM993072.1 |
| 40 | *Fritillaria* | *F. forbesii* | 1 | / | LM993073.1 |
| 41 | *Fritillaria* | *F. frankiorum* | 1 | / | LM993074.1 |
| 42 | *Fritillaria* | *F. fusca* | 1 | / | MW890003.1 |
| 43 | *Fritillaria* | *F. gentneri* | 1 | / | AY624437.1 |
| 44 | *Fritillaria* | *F. gibbosa* | 3 | / | AY624438.1, LM993075.1, KM085605.1 |
| 45 | *Fritillaria* | *F. glauca* | 1 | / | AY624439.1 |
| 46 | *Fritillaria* | *F. graeca* | 1 | / | LM993076.1 |
| 47 | *Fritillaria* | *F. gussichiae* | 1 | / | LM993077.1 |
| 48 | *Fritillaria* | *F. hermonis* | 1 | / | AY624440.1 |
| 49 | *Fritillaria* | *F. hupehensis* | 10 | 10 | GQ205143.1, KF906216.1, KF906217.1, KF906218.1, KF906219.1, KF906220.1, KF712486.1, MN811001.1, MN811002.1, MN811003.1 |
| 50 | *Fritillaria* | *F. imperialis* | 2 | / | AY624441.1, KP998198.1 |
| 51 | *Fritillaria* | *F. involucrata* | 3 | / | LM993078.1, LM993079.1, MN917988.1 |
| 52 | *Fritillaria* | *F. japonica* | 4 | / | AY624442.1, LM993080.1, KM085607.1, KP998199.1 |
| 53 | *Fritillaria* | *F. kaiensis* | 1 | / | LM993081.1 |
| 54 | *Fritillaria* | *F. karelinii* | 8 | / | LM993082.1, KX354691.1, MG211818.1, MG211821.1, MG946170.1, MG946171.1, MG946172.1, MZ970132.1 |
| 55 | *Fritillaria* | *F. koidzumiana* | 4 | / | AB024390.1, AB034939.1, LM993083.1, KP998200.1 |
| 56 | *Fritillaria* | *F. kotschyana* | 2 | / | JN417342.1, LM993084.1 |
| 57 | *Fritillaria* | *F. kurdica* | 1 | / | LM993063.1 |
| 58 | *Fritillaria* | *F. latakiensis* | 1 | / | LM993085.1 |
| 59 | *Fritillaria* | *F. latifolia* | 1 | / | LM993086.1 |
| 60 | *Fritillaria* | *F. liliacea* | 1 | / | LM993087.1 |
| 61 | *Fritillaria* | *F. lusitanica* | 1 | / | AY624443.1 |
| 62 | *Fritillaria* | *F. maximowiczii* | 11 | / | AY624444.1, LM993088.1, KP711866.1, MG525331.1, MK258138.1, MN810992.1, MN810993.1, MN810994.1, OQ267636.1, OQ267637.1, OQ267638.1 |
| 63 | *Fritillaria* | *F. meleagris* | 4 | / | AY624445.1, KM085608.1, MF543520.1, MN917989.1 |
| 64 | *Fritillaria* | *F. meleagroides* | 4 | / | LM993089.1, MF947710.1, MG946173.1, MG946174.1 |
| 65 | *Fritillaria* | *F. mellea* | 2 | / | GQ205144.1, GQ205145.1 |
| 66 | *Fritillaria* | *F. messanensis subsp. messanensis* | 1 | / | LM993090.1 |
| 67 | *Fritillaria* | *F. michailovskyi* | 1 | / | AY624446.1 |
| 68 | *Fritillaria* | *F. micrantha* | 3 | / | AY624447.1, LM993091.1, KM085609.1 |
| 69 | *Fritillaria* | *F. minuta* | 1 | / | AY624448.1 |
| 70 | *Fritillaria* | *F. monantha* | 9 | / | KF906212.1, KF906213.1, KF906214.1, KF906215.1, MG525337.1, MK258143.1, MN810974.1, MN810975.1, MN810976.1 |
| 71 | *Fritillaria* | *F. montana* | 4 | / | LM993092.1, LM993093.1, MF543521.1, MF543522.1 |
| 72 | *Fritillaria* | *F. muraiana* | 2 | / | LM993094.1, LM993095.1 |
| 73 | *Fritillaria* | *F. mutabilis* | 1 | / | LM993096.1 |
| 74 | *Fritillaria* | *F. obliqua subsp. obliqua* | 1 | / | LM993097.1 |
| 75 | *Fritillaria* | *F. obliqua subsp. tuntasia* | 2 | / | LM993098.1, LM993099.1 |
| 76 | *Fritillaria* | *F. olgae* | 1 | / | MW081400.1 |
| 77 | *Fritillaria* | *F. olivieri* | 1 | / | AY624449.1 |
| 78 | *Fritillaria* | *F. omeiensis* | 3 | / | MN810995.1, MN810996.1, MN810997.1 |
| 79 | *Fritillaria* | *F. oranensis* | 1 | / | LM993100.1 |
| 80 | *Fritillaria* | *F. orientalis* | 2 | / | LM993101.1, LM993102.1 |
| 81 | *Fritillaria* | *F. pallidiflora* | 7 | 10 | AY624450.1, MG211822.1, MG946175.1, MG946176.1, MH593364.1, MH593365.1, MH593366.1 |
| 82 | *Fritillaria* | *F. persica* | 3 | / | AY624451.1, KM085610.1, MF947709.1 |
| 83 | *Fritillaria* | *F. phaeanthera* | 1 | / | AY624452.1 |
| 84 | *Fritillaria* | *F. pinardii* | 1 | / | LM993103.1 |
| 85 | *Fritillaria* | *F. pluriflora* | 1 | / | LM993104.1 |
| 86 | *Fritillaria* | *F. pontica* | 1 | / | LM993106.1 |
| 87 | *Fritillaria* | *F. przewalskii* | 11 | 20 | GQ205134.1, KF906227.1, KY884661.1, MG525342.1, MG946177.1, MH244908.1, MK258142.1, MH593347.1, MH593348.1, MH593349.1, MT806747.1 |
| 88 | *Fritillaria* | *F. pudica* | 4 | / | AY624453.1, LM993107.1, KM085611.1, KX676942.1 |
| 89 | *Fritillaria* | *F. puqiensis* | 1 | / | KY884659.1 |
| 90 | *Fritillaria* | *F. pyrenaica subsp. pyrenaica* | 1 | / | LM993108.1 |
| 91 | *Fritillaria* | *F. qimenensis* | 1 | / | KY884657.1 |
| 92 | *Fritillaria* | *F. raddeana* | 1 | / | AY624454.1 |
| 93 | *Fritillaria* | *F. recurva* | 1 | / | AY624455.1 |
| 94 | *Fritillaria* | *F. reuteri* | 1 | / | AY624456.1 |
| 95 | *Fritillaria* | *F. rixii* | 1 | / | LM993109.1 |
| 96 | *Fritillaria* | *F. ruthenica* | 1 | / | LM993110.1 |
| 97 | *Fritillaria* | *F. sewerzowii* | 4 | / | AY624457.1, LM993111.1, LM993112.1, KM085612.1 |
| 98 | *Fritillaria* | *F. shikokiana* | 2 | / | LM993113.1, LM993114.1 |
| 99 | *Fritillaria* | *F. sibthorpiana subsp. enginiana* | 1 | / | LM993115.1 |
| 100 | *Fritillaria* | *F. sibthorpiana subsp. sibthorpiana* | 1 | / | LM993116.1 |
| 101 | *Fritillaria* | *F. sichuanica* | 7 | / | KP711867.1, MG525347.1, MH244907.1, MK258141.1, MT806748.1, MN810967.1, MN810968.1 |
| 102 | *Fritillaria* | *F. sinica* | 3 | / | KF906226.1, MH244912.1, MN810971.1 |
| 103 | *Fritillaria* | *F. sororum* | 2 | / | LM993117.1, LM993118.1 |
| 104 | *Fritillaria* | *F. stenanthera* | 1 | / | LM993119.1 |
| 105 | *Fritillaria* | *F. stribrnyi* | 1 | / | LM993120.1 |
| 106 | *Fritillaria* | *F. sulcisquamosa* | 1 | / | GQ205150.1 |
| 107 | *Fritillaria* | *F. taipaiensis* | 22 | 20 | KC543997.1, KC713822.1, KC713823.1, KF769144.1, KP711868.1, MG525344.1, MH659893.1, MH244910.1, MH593356.1, MH593357.1, MH593358.1, MH593359.1, MK642356.1, MT806749.1, MT806750.1, MT806751.1, MT806752.1, MT806753.1, MT806754.1, OQ411258.1, OQ433930.1, OQ433931.1 |
| 108 | *Fritillaria* | *F. tenella* | 1 | / | AY624458.1 |
| 109 | *Fritillaria* | *F. theophrasti* | 1 | / | LM993121.1 |
| 110 | *Fritillaria* | *F. thessala subsp. reiseri* | 1 | / | LM993122.1 |
| 111 | *Fritillaria* | *F. thessala subsp. thessala* | 1 | / | LM993123.1 |
| 112 | *Fritillaria* | *F. thunbergii* | 16 | 10 | AB049527.1, GQ205146.1, JQ724626.1, KF850817.1, LM993124.1, KP711869.1, KY646165.1, KY884653.1, KY884654.1, KY884655.1, KY884656.1, MG525336.1, MH244914.1, MH593360.1, MH593361.1, MH593362.1 |
| 113 | *Fritillaria* | *F. thunbergii var. chekiangensis* | 1 | / | MG525339.1 |
| 114 | *Fritillaria* | *F. tokushimensis* | 1 | / | LM993125.1 |
| 115 | *Fritillaria* | *F. tortifolia* | 11 | / | LM993126.1, KP711870.1, MG211819.1, MG525334.1, MG946178.1, MG946179.1, MG946180.1, MG946181.1, MN810985.1, MN810986.1, MN810987.1 |
| 116 | *Fritillaria* | *F. tubiformis* | 3 | / | AY624459.1, MN917993.1, MN917994.1 |
| 117 | *Fritillaria* | *F. tubiformis subsp. moggridgei* | 3 | / | MN917990.1, MN917991.1, MN917992.1 |
| 118 | *Fritillaria* | *F. tubiformis subsp. tubiformis* | 1 | / | LM993127.1 |
| 119 | *Fritillaria* | *F. tubiformis var. burnatii* | 2 | / | MN917986.1, MN917987.1 |
| 120 | *Fritillaria* | *F. unibracteata* | 12 | 20 | GQ205142.1, GQ205149.1, KP711871.1, KP711872.1, MG525349.1, MG946182.1, MH244909.1, MK258140.1, MH593350.1, MH593351.1, MH593352.1, OM807063.1 |
| 121 | *Fritillaria* | *F. unibracteata var. longinectarea* | 6 | / | GQ205135.1, GQ205138.1, KF906225.1, MG525350.1, OM807060.1, OM807061.1 |
| 122 | *Fritillaria* | *F. unibracteata var. maculata* | 1 | / | OM807062.1 |
| 123 | *Fritillaria* | *F. unibracteata var. wabuensis* | 2 | 20 | GQ205147.1, KF769142.1 |
| 124 | *Fritillaria* | *F. usuriensis* | 10 | 10 | JN417343.1, LM993128.1, LM993129.1, KM085613.1, KY646166.1, MG946183.1, MH593367.1, MH593368.1, MH593369.1, MT261156.1 |
| 125 | *Fritillaria* | *F. uva-vulpis* | 1 | / | LM993130.1 |
| 126 | *Fritillaria* | *F. verticillata* | 6 | / | LM993131.1, KP711873.1, MG211823.1, MG525335.1, MG946184.1, MG946185.1 |
| 127 | *Fritillaria* | *F. walujewii* | 13 | 10 | LM993132.1, LM993133.1, KP711874.1, MG211820.1, MG525333.1, MG946186.1, MG946187.1, MG946188.1, MG946189.1, MN810988.1, MN810989.1, MN810990.1, MN810991.1 |
| 128 | *Fritillaria* | *F. wanjiangensis* | 1 | / | KY884658.1 |
| 129 | *Fritillaria* | *F. whittallii* | 1 | / | LM993134.1 |
| 130 | *Fritillaria* | *F. yuminensis* | 4 | / | MG200070.1, MG946190.1, MG946191.1, MG946192.1 |
| 131 | *Fritillaria* | *F. yuzhongensis* | 5 | / | MG525343.1, MH244911.1, MK258139.1, MN810969.1, MN810970.1 |
| 132 | *Fritillaria* | *F. zagrica* | 1 | / | LM993135.1 |

Supplementary Table 4 Candidate SNPs in Fritillariae Cirrhosae Bulbus ITS and *mat*K sequences

| NO. | Genus | Species | ITS 341 | *matK* 336 |
| --- | --- | --- | --- | --- |
| 1 | *Fritillaria* | *F. cirrhosa* | T | A |
| 2 | *Fritillaria* | *F. delavayi* | T | A |
| 3 | *Fritillaria* | *F. przewalskii* | T | A |
| 4 | *Fritillaria* | *F. taipaiensis* | T | A |
| 5 | *Fritillaria* | *F. unibracteata* | T | A |
| 6 | *Fritillaria* | *F. unibracteata.var. wabuensis* | T | A |
| 7 | *Fritillaria* | *F. hupehensis* | C | G |
| 8 | *Fritillaria* | *F. thunbergii* | C | G |
| 9 | *Fritillaria* | *F. ussuriensis* | C | G |
| 10 | *Fritillaria* | *F. pallidiflora* | C | G |
| 11 | *Fritillaria* | *F.walujewii* | C | G |
| 12 | *Fritillaria* | *F. acmopetala* | C | G |
| 13 | *Fritillaria* | *F. affinis* | C | G |
| 14 | *Fritillaria* | *F. agrestis* | C | G |
| 15 | *Fritillaria* | *F. alburyana* | C | G |
| 16 | *Fritillaria* | *F. anhuiensis* | C | G |
| 17 | *Fritillaria* | *F. atropurpurea* | C | G |
| 18 | *Fritillaria* | *F. aurea* | C | G |
| 19 | *Fritillaria* | *F. biflora* | C | G |
| 20 | *Fritillaria* | *F. borealis* | C | G |
| 21 | *Fritillaria* | *F. brandegeei* | C | G |
| 22 | *Fritillaria* | *F. camtschatcensis* | C | G |
| 23 | *Fritillaria* | *F. camschatcensis* | C | G |
| 24 | *Fritillaria* | *F. caucasica* | C | G |
| 25 | *Fritillaria* | *F. charybdae* | C | G |
| 26 | *Fritillaria* | *F. chitralensis* | C | G |
| 27 | *Fritillaria* | *F. crassicaulis* | C | G |
| 28 | *Fritillaria* | *F. crassifolia* | C | G |
| 29 | *Fritillaria* | *F. dagana* | C | G |
| 30 | *Fritillaria* | *F. dajinensis* | C | G |
| 31 | *Fritillaria* | *F. davidi* | C | G |
| 32 | *Fritillaria* | *F. davidii* | C | G |
| 33 | *Fritillaria* | *F. eastwoodiae* | C | G |
| 34 | *Fritillaria* | *F. ebeiensis* | C | G |
| 35 | *Fritillaria* | *F. falcata* | C | G |
| 36 | *Fritillaria* | *F. ferganensis* | C | G |
| 37 | *Fritillaria* | *F. formica* | C | G |
| 38 | *Fritillaria* | *F. gentneri* | C | G |
| 39 | *Fritillaria* | *F. gibbosa* | C | G |
| 40 | *Fritillaria* | *F. glauca* | C | G |
| 41 | *Fritillaria* | *F. gracilis* | C | G |
| 42 | *Fritillaria* | *F. haplostoma* | C | G |
| 43 | *Fritillaria* | *F. hermontis* | C | G |
| 44 | *Fritillaria* | *F. imperialis* | C | G |
| 45 | *Fritillaria* | *F. involucrata* | C | G |
| 46 | *Fritillaria* | *F. japonica* | C | G |
| 47 | *Fritillaria* | *F. karelini* | C | G |
| 48 | *Fritillaria* | *F. karelinii* | C | G |
| 49 | *Fritillaria* | *F. koidzumiana* | C | G |
| 50 | *Fritillaria* | *F. kotschyana* | C | G |
| 51 | *Fritillaria* | *F. latifolia* | C | G |
| 52 | *Fritillaria* | *F. liliacea* | C | G |
| 53 | *Fritillaria* | *F. lusitanica* | C | G |
| 54 | *Fritillaria* | *F. maximoviczii* | C | G |
| 55 | *Fritillaria* | *F. maximowiczii* | C | G |
| 56 | *Fritillaria* | *F. meleagris* | C | G |
| 57 | *Fritillaria* | *F. meleagroides* | C | G |
| 58 | *Fritillaria* | *F. mellea* | C | G |
| 59 | *Fritillaria* | *F. messanensis* | C | G |
| 60 | *Fritillaria* | *F. michailovskyi* | C | G |
| 61 | *Fritillaria* | *F. micrantha* | C | G |
| 62 | *Fritillaria* | *F. minuta* | C | G |
| 63 | *Fritillaria* | *F. monantha* | C | G |
| 64 | *Fritillaria* | *F. montana* | C | G |
| 65 | *Fritillaria* | *F. ojaiensis* | C | G |
| 66 | *Fritillaria* | *F. olivieri* | C | G |
| 67 | *Fritillaria* | *F. omeiensis* | C | G |
| 68 | *Fritillaria* | *F. pellucida* | C | G |
| 69 | *Fritillaria* | *F. persica* | C | G |
| 70 | *Fritillaria* | *F. phaeanthera* | C | G |
| 71 | *Fritillaria* | *F. pinetorum* | C | G |
| 72 | *Fritillaria* | *F. pluriflora* | C | G |
| 73 | *Fritillaria* | *F. polaris* | C | G |
| 74 | *Fritillaria* | *F. pudica* | C | G |
| 75 | *Fritillaria* | *F. puqiensis* | C | G |
| 76 | *Fritillaria* | *F. purdyi* | C | G |
| 77 | *Fritillaria* | *F. qimenensis* | C | G |
| 78 | *Fritillaria* | *F. raddeana* | C | G |
| 79 | *Fritillaria* | *F. ragusina* | C | G |
| 80 | *Fritillaria* | *F. recurva* | C | G |
| 81 | *Fritillaria* | *F. reuteri* | C | G |
| 82 | *Fritillaria* | *F. roderickii* | C | G |
| 83 | *Fritillaria* | *F. sewerzowii* | C | G |
| 84 | *Fritillaria* | *F. sichuanica* | C | G |
| 85 | *Fritillaria* | *F. sinica* | C | G |
| 86 | *Fritillaria* | *F. sonnikovae* | C | G |
| 87 | *Fritillaria* | *F. sp.* | C | G |
| 88 | *Fritillaria* | *F. stenanthera* | C | G |
| 89 | *Fritillaria* | *F. straussii* | C | G |
| 90 | *Fritillaria* | *F. striata* | C | G |
| 91 | *Fritillaria* | *F. sulcisquamosa* | C | G |
| 92 | *Fritillaria* | *F. tenella* | C | G |
| 93 | *Fritillaria* | *F. tortifolia* | C | G |
| 94 | *Fritillaria* | *F. tubiformis* | C | G |
| 95 | *Fritillaria* | *F. unibracteata var. longinectarea* | C | G |
| 96 | *Fritillaria* | *F. urticans* | C | G |
| 97 | *Fritillaria* | *F. venusta* | C | G |
| 98 | *Fritillaria* | *F. verticillata* | C | G |
| 99 | *Fritillaria* | *F. viridea* | C | G |
| 100 | *Fritillaria* | *F. wanjiangensis* | C | G |
| 101 | *Fritillaria* | *F. yuminensis* | C | G |
| 102 | *Fritillaria* | *F. yuzhongensis* | C | G |
| 103 | *Fritillaria* | *F. zagrica* | C | G |

Supplementary Table 5 Candidate SNPs in ITS and *mat*K sequences for individual adulterated species

| NO. | Genus | Species | ITS 361 | ITS 366 | *mat*K 923 | *mat*K 1173 |
| --- | --- | --- | --- | --- | --- | --- |
| 1 | *Fritillaria* | *F. cirrhosa* | T | A | G | T |
| 2 | *Fritillaria* | *F. delavayi* | T | A | G | T |
| 3 | *Fritillaria* | *F. przewalskii* | T | A | G | T |
| 4 | *Fritillaria* | *F. taipaiensis* | T | A | G | T |
| 5 | *Fritillaria* | *F. unibracteata* | T | A | G | T |
| 6 | *Fritillaria* | *F. unibracteata.var. wabuensis* | T | A | G | T |
| 7 | *Fritillaria* | *F. hupehensis* | T | A | A | T |
| 8 | *Fritillaria* | *F. thunbergii* | C | A | G | T |
| 9 | *Fritillaria* | *F. ussuriensis* | T | G | G | T |
| 10 | *Fritillaria* | *F. pallidiflora* | T | A | G | A |
| 11 | *Fritillaria* | *F.walujewii* | T | A | G | A |
| 12 | *Fritillaria* | *F. affinis* | T | A | G | T |
| 13 | *Fritillaria* | *F. anhuiensis* | T | A | G | T |
| 14 | *Fritillaria* | *F. camschatcensis* | T | A | G | T |
| 15 | *Fritillaria* | *F. caucasica* | T | A | G | T |
| 16 | *Fritillaria* | *F. crassicaulis* | T | A | G | T |
| 17 | *Fritillaria* | *F. dagana* | T | A | G | T |
| 18 | *Fritillaria* | *F. dajinensis* | T | A | G | T |
| 19 | *Fritillaria* | *F. davidii* | T | A | G | T |
| 20 | *Fritillaria* | *F. gibbosa* | T | A | G | T |
| 21 | *Fritillaria* | *F. imperialis* | T | A | G | T |
| 22 | *Fritillaria* | *F. karelinii* | T | A | G | T |
| 23 | *Fritillaria* | *F. maximowiczii* | T | A | G | T |
| 24 | *Fritillaria* | *F. meleagris* | T | A | G | T |
| 25 | *Fritillaria* | *F. meleagroides* | T | A | G | T |
| 26 | *Fritillaria* | *F. micrantha* | T | A | G | T |
| 27 | *Fritillaria* | *F. monantha* | T | A | G | T |
| 28 | *Fritillaria* | *F. montana* | T | A | G | T |
| 29 | *Fritillaria* | *F. olivieri* | T | A | G | T |
| 30 | *Fritillaria* | *F. omeiensis* | T | A | G | T |
| 31 | *Fritillaria* | *F. persica* | T | A | G | T |
| 32 | *Fritillaria* | *F. raddeana* | T | A | G | T |
| 33 | *Fritillaria* | *F. reuteri* | T | A | G | T |
| 34 | *Fritillaria* | *F. sichuanica* | T | A | G | T |
| 35 | *Fritillaria* | *F. sinica* | T | A | G | T |
| 36 | *Fritillaria* | *F. tortifolia* | T | A | G | T |
| 37 | *Fritillaria* | *F. verticillata* | T | A | G | T |
| 38 | *Fritillaria* | *F. yuminensis* | T | A | G | T |
| 39 | *Fritillaria* | *F. yuzhongensis* | T | A | G | T |
| 40 | *Fritillaria* | *F. zagrica* | T | A | G | T |
| 41 | *Fritillaria* | *F. acmopetala* | T | A | G | T |
| 42 | *Fritillaria* | *F. agrestis* | T | A | G | T |
| 43 | *Fritillaria* | *F. alburyana* | T | A | G | T |
| 44 | *Fritillaria* | *F. atropurpurea* | T | A | G | T |
| 45 | *Fritillaria* | *F. aurea* | T | A | G | T |
| 46 | *Fritillaria* | *F. chitralensis* | T | A | G | T |
| 47 | *Fritillaria* | *F. eastwoodiae* | T | A | G | T |
| 48 | *Fritillaria* | *F. falcata* | T | A | G | T |
| 49 | *Fritillaria* | *F. involucrata* | T | A | G | T |
| 50 | *Fritillaria* | *F. japonica* | T | A | G | T |
| 51 | *Fritillaria* | *F. karelini* | T | A | G | T |
| 52 | *Fritillaria* | *F. koidzumiana* | T | A | G | T |
| 53 | *Fritillaria* | *F. kotschyana* | T | A | G | T |
| 54 | *Fritillaria* | *F. latifolia* | T | A | G | T |
| 55 | *Fritillaria* | *F. liliacea* | T | A | G | T |
| 56 | *Fritillaria* | *F. lusitanica* | T | A | G | T |
| 57 | *Fritillaria* | *F. michailovskyi* | T | A | G | T |
| 58 | *Fritillaria* | *F. minuta* | T | A | G | T |
| 59 | *Fritillaria* | *F. pluriflora* | T | A | G | T |
| 60 | *Fritillaria* | *F. pudica* | T | A | G | T |
| 61 | *Fritillaria* | *F. recurva* | T | A | G | T |
| 62 | *Fritillaria* | *F. stenanthera* | T | A | G | T |
| 63 | *Fritillaria* | *F. stenanthera* | T | A | G | T |
| 64 | *Fritillaria* | *F. crassifolia* | T | A | / | / |
| 65 | *Fritillaria* | *F. mellea* | T | A | / | / |
| 66 | *Fritillaria* | *F. sonnikovae* | T | A | / | / |
| 67 | *Fritillaria* | *F. straussii* | T | A | / | / |
| 68 | *Fritillaria* | *F. biflora* | T | A | / | / |
| 69 | *Fritillaria* | *F. borealis* | T | A | / | / |
| 70 | *Fritillaria* | *F. brandegeei* | T | A | / | / |
| 71 | *Fritillaria* | *F. camtschatcensis* | T | A | / | / |
| 72 | *Fritillaria* | *F. charybdae* | T | A | / | / |
| 73 | *Fritillaria* | *F. davidi* | T | A | / | / |
| 74 | *Fritillaria* | *F. ebeiensis* | T | A | / | / |
| 75 | *Fritillaria* | *F. ferganensis* | T | A | / | / |
| 76 | *Fritillaria* | *F. formica* | T | A | / | / |
| 77 | *Fritillaria* | *F. gentneri* | T | A | / | / |
| 78 | *Fritillaria* | *F. glauca* | T | A | / | / |
| 79 | *Fritillaria* | *F. gracilis* | T | A | / | / |
| 80 | *Fritillaria* | *F. haplostoma* | T | A | / | / |
| 81 | *Fritillaria* | *F. hermontis* | T | A | / | / |
| 82 | *Fritillaria* | *F. maximoviczii* | T | A | / | / |
| 83 | *Fritillaria* | *F. messanensis* | T | A | / | / |
| 84 | *Fritillaria* | *F. ojaiensis* | T | A | / | / |
| 85 | *Fritillaria* | *F. pellucida* | T | A | / | / |
| 86 | *Fritillaria* | *F. phaeanthera* | T | A | / | / |
| 87 | *Fritillaria* | *F. pinetorum* | T | A | / | / |
| 88 | *Fritillaria* | *F. polaris* | T | A | / | / |
| 89 | *Fritillaria* | *F. puqiensis* | T | A | / | / |
| 90 | *Fritillaria* | *F. purdyi* | T | A | / | / |
| 91 | *Fritillaria* | *F. qimenensis* | T | A | / | / |
| 92 | *Fritillaria* | *F. ragusina* | T | A | / | / |
| 93 | *Fritillaria* | *F. roderickii* | T | A | / | / |
| 94 | *Fritillaria* | *F. sp.* | T | A | / | / |
| 95 | *Fritillaria* | *F. striata* | T | A | / | / |
| 96 | *Fritillaria* | *F. sulcisquamosa* | T | A | / | / |
| 97 | *Fritillaria* | *F. tenella* | T | A | / | / |
| 98 | *Fritillaria* | *F. tubiformis* | T | A | / | / |
| 99 | *Fritillaria* | *F. urticans* | T | A | / | / |
| 100 | *Fritillaria* | *F. venusta* | T | A | / | / |
| 101 | *Fritillaria* | *F. viridea* | T | A | / | / |
| 102 | *Fritillaria* | *F. wanjiangensis* | T | A | / | / |
| 103 | *Fritillaria* | *F. crassifolia subsp.poluninii* | / | / | G | T |
| 104 | *Fritillaria* | *F. eduardii* | / | / | G | T |
| 105 | *Fritillaria* | *F. fusca* | / | / | G | T |
| 106 | *Fritillaria* | *F. pinardii* | / | / | G | T |
| 107 | *Fritillaria* | *F. unibracteata var.longinectarea* | / | / | G | T |
| 108 | *Fritillaria* | *F. unibracteata var.maculata* | / | / | G | T |
| 109 | *Fritillaria* | *F. uva-vulpis* | / | / | G | T |
| 110 | *Fritillaria* | *F. acmopetala subsp.acmopetala* | / | / | G | T |
| 111 | *Fritillaria* | *F. alfredae subsp.glaucoviridis* | / | / | G | T |
| 112 | *Fritillaria* | *F. amabilis* | / | / | G | T |
| 113 | *Fritillaria* | *F. amana* | / | / | G | T |
| 114 | *Fritillaria* | *F. armena* | / | / | G | T |
| 115 | *Fritillaria* | *F. assyriaca subsp.assyriaca* | / | / | G | T |
| 116 | *Fritillaria* | *F. ayakoana* | / | / | G | T |
| 117 | *Fritillaria* | *F. bithynica* | / | / | G | T |
| 118 | *Fritillaria* | *F. bucharica* | / | / | G | T |
| 119 | *Fritillaria* | *F. carica* | / | / | G | T |
| 120 | *Fritillaria* | *F. conica* | / | / | G | T |
| 121 | *Fritillaria* | *F. crassifolia subsp.crassifolia* | / | / | G | T |
| 122 | *Fritillaria* | *F. davisii* | / | / | G | T |
| 123 | *Fritillaria* | *F. drenovskii* | / | / | G | T |
| 124 | *Fritillaria* | *F. ehrhartii* | / | / | G | T |
| 125 | *Fritillaria* | *F. elwesii* | / | / | G | T |
| 126 | *Fritillaria* | *F. fleischeriana* | / | / | G | T |
| 127 | *Fritillaria* | *F. forbesii* | / | / | G | T |
| 128 | *Fritillaria* | *F. frankiorum* | / | / | G | T |
| 129 | *Fritillaria* | *F. graeca* | / | / | G | T |
| 130 | *Fritillaria* | *F. gussichiae* | / | / | G | T |
| 131 | *Fritillaria* | *F. kurdica* | / | / | G | T |
| 132 | *Fritillaria* | *F. latakiensis* | / | / | G | T |
| 133 | *Fritillaria* | *F. meleagris subsp.meleagris* | / | / | G | T |
| 134 | *Fritillaria* | *F. messanensis subsp.messanensis* | / | / | G | T |
| 135 | *Fritillaria* | *F. muraiana* | / | / | G | T |
| 136 | *Fritillaria* | *F. mutabilis* | / | / | G | T |
| 137 | *Fritillaria* | *F. obliqua subsp.obliqua* | / | / | G | T |
| 138 | *Fritillaria* | *F. obliqua subsp.tuntasia* | / | / | G | T |
| 139 | *Fritillaria* | *F. oranensis* | / | / | G | T |
| 140 | *Fritillaria* | *F. orientalis* | / | / | G | T |
| 141 | *Fritillaria* | *F. pontica* | / | / | G | T |
| 142 | *Fritillaria* | *F. pyrenaica subsp.pyrenaica* | / | / | G | T |
| 143 | *Fritillaria* | *F. rixii* | / | / | G | T |
| 144 | *Fritillaria* | *F. ruthenica* | / | / | G | T |
| 145 | *Fritillaria* | *F. shikokiana* | / | / | G | T |
| 146 | *Fritillaria* | *F. sibthorpiana subsp.enginiana* | / | / | G | T |
| 147 | *Fritillaria* | *F. sororum* | / | / | G | T |
| 148 | *Fritillaria* | *F. stribrnyi* | / | / | G | T |
| 149 | *Fritillaria* | *F. theophrasti* | / | / | G | T |
| 150 | *Fritillaria* | *F. thessala subsp.reiseri* | / | / | G | T |
| 151 | *Fritillaria* | *F. thessala subsp.thessala* | / | / | G | T |
| 152 | *Fritillaria* | *F. thunbergi ivar.chekiangensis* | / | / | G | T |
| 153 | *Fritillaria* | *F. tokushimensis* | / | / | G | T |
| 154 | *Fritillaria* | *F. tubiformis subsp.tubiformis* | / | / | G | T |
| 155 | *Fritillaria* | *F. whittallii* | / | / | G | T |

Supplementary Table 6 Detailed BLAST results with the score being max and total

| SNP sites | Input Sequence | Objective species | Organism | Whether was unique specie in Blast of NCBI? |
| --- | --- | --- | --- | --- |
| ITS 341 | GAGCACCAGCAGGATGT**T**GTGGCCCCCTGTCGCCTTAAGG | Fritillaria Cirrhosae Bulbus | *F. cirrhosa* | Yes |
|  |  |  | *F. delavayi* | Yes |
|  |  |  | *F. przewalskii* | Yes |
|  |  |  | *F. unibracteata* | Yes |
|  |  |  | *F. unibracteata var. wabuensis* | Yes |
|  |  |  | *F. taipaiensis* | Yes |
| ITS 366 | TCGTGGCCCCCCGTCGCCTTAAGGGGCTCAAG**G**GACCCGG | *F. ussuriensis* | *F. ussuriensis* | Yes |
| ITS 361 | TCGTGGCCCCCCGTCGCCTTAAGGGGC**C**CAAGAGACCCGG | *F. thunbergii* | *F. thunbergii* | Yes |
| *mat*K 923 | TCGCATTCAGATACATAAGAATTGTAGGAACCAAAATA**A**T | *F. hupehensis* | *F. hupehensis* | Yes |
| *mat*K 1173 | ATCCTAAATTCTGAGATTT**A**GGTATTTCTTTTTCTTCGGG | Fritillaria Pallidiflorae Bulbus | *F. pallidiflora* | Yes |
|  |  |  | *F. walujewii* | Yes |

Supplementary Table 7 The raw data of all proportions for Linearity (n=3)

| NO. | Proportion of adulteration (C%) | Authentic *F. cirrhosa* (T%) | Adulterated *F. hupehensis* (C%) |
| --- | --- | --- | --- |
| 1 | 1% | 99% | 1% |
| 2 | 1% | 97% | 3% |
| 3 | 1% | 100% | 0% |
| 4 | 2% | 98% | 2% |
| 5 | 2% | 98% | 2% |
| 6 | 2% | 98% | 2% |
| 7 | 4% | 96% | 4% |
| 8 | 4% | 96% | 4% |
| 9 | 4% | 96% | 4% |
| 10 | 6% | 94% | 6% |
| 11 | 6% | 94% | 6% |
| 12 | 6% | 94% | 6% |
| 13 | 8% | 92% | 8% |
| 14 | 8% | 92% | 8% |
| 15 | 8% | 92% | 8% |
| 16 | 10% | 90% | 10% |
| 17 | 10% | 90% | 10% |
| 18 | 10% | 90% | 10% |
| 19 | 20% | 81% | 19% |
| 20 | 20% | 80% | 20% |
| 21 | 20% | 80% | 20% |
| 22 | 30% | 70% | 30% |
| 23 | 30% | 70% | 30% |
| 24 | 30% | 30% | 30% |
| 25 | 40% | 60% | 40% |
| 26 | 40% | 60% | 40% |
| 27 | 40% | 60% | 40% |
| 28 | 50% | 50% | 50% |
| 29 | 50% | 50% | 50% |
| 30 | 50% | 51% | 49% |

Supplementary Table 8 The raw data of all proportions for LOD/LOQ (n=20)

| NO. | Proportion of adulteration (C%) | Authentic *F. cirrhosa* (T%) | Adulterated *F. hupehensis* (C%) |
| --- | --- | --- | --- |
| 1 | 1% | 100% | 0% |
| 2 | 1% | 99% | 1% |
| 3 | 1% | 99% | 1% |
| 4 | 1% | 95% | 5% |
| 5 | 1% | 101% | -1% |
| 6 | 1% | 100% | 0% |
| 7 | 1% | 99% | 1% |
| 8 | 1% | 96% | 4% |
| 9 | 1% | 98% | 2% |
| 10 | 1% | 93% | 3% |
| 11 | 1% | 102% | -2% |
| 12 | 1% | 102% | -2% |
| 13 | 1% | 94% | 6% |
| 14 | 1% | 97% | 3% |
| 15 | 1% | 100% | 0% |
| 16 | 1% | 99% | 1% |
| 17 | 1% | 99% | 1% |
| 18 | 1% | 97% | 3% |
| 19 | 1% | 96% | 4% |
| 20 | 1% | 96% | 4% |
| 21 | 2% | 98% | 2% |
| 22 | 2% | 98% | 2% |
| 23 | 2% | 98% | 2% |
| 24 | 2% | 98% | 2% |
| 25 | 2% | 98% | 2% |
| 26 | 2% | 98% | 2% |
| 27 | 2% | 98% | 2% |
| 28 | 2% | 98% | 2% |
| 29 | 2% | 97% | 3% |
| 30 | 2% | 98% | 2% |
| 31 | 2% | 98% | 2% |
| 32 | 2% | 98% | 2% |
| 33 | 2% | 98% | 2% |
| 34 | 2% | 98% | 2% |
| 35 | 2% | 98% | 2% |
| 36 | 2% | 98% | 2% |
| 37 | 2% | 98% | 2% |
| 38 | 2% | 98% | 2% |
| 39 | 2% | 98% | 2% |
| 40 | 2% | 98% | 2% |
| 41 | 4% | 96% | 4% |
| 42 | 4% | 95% | 5% |
| 43 | 4% | 96% | 4% |
| 44 | 4% | 95% | 5% |
| 45 | 4% | 97% | 3% |
| 46 | 4% | 96% | 4% |
| 47 | 4% | 96% | 4% |
| 48 | 4% | 96% | 4% |
| 49 | 4% | 96% | 4% |
| 50 | 4% | 96% | 4% |
| 51 | 4% | 96% | 4% |
| 52 | 4% | 96% | 4% |
| 53 | 4% | 96% | 4% |
| 54 | 4% | 96% | 4% |
| 55 | 4% | 96% | 4% |
| 56 | 4% | 96% | 4% |
| 57 | 4% | 96% | 4% |
| 58 | 4% | 96% | 4% |
| 59 | 4% | 96% | 4% |
| 60 | 4% | 96% | 4% |
| 61 | 6% | 95% | 5% |
| 62 | 6% | 94% | 6% |
| 63 | 6% | 93% | 7% |
| 64 | 6% | 94% | 6% |
| 65 | 6% | 94% | 6% |
| 66 | 6% | 94% | 6% |
| 67 | 6% | 94% | 6% |
| 68 | 6% | 94% | 6% |
| 69 | 6% | 94% | 6% |
| 70 | 6% | 93% | 7% |
| 71 | 6% | 94% | 6% |
| 72 | 6% | 95% | 5% |
| 73 | 6% | 94% | 6% |
| 74 | 6% | 94% | 6% |
| 75 | 6% | 94% | 6% |
| 76 | 6% | 94% | 6% |
| 77 | 6% | 94% | 6% |
| 78 | 6% | 94% | 6% |
| 79 | 6% | 94% | 6% |
| 80 | 6% | 94% | 6% |
| 81 | 8% | 91% | 9% |
| 82 | 8% | 91% | 9% |
| 83 | 8% | 91% | 9% |
| 84 | 8% | 91% | 9% |
| 85 | 8% | 91% | 9% |
| 86 | 8% | 92% | 8% |
| 87 | 8% | 92% | 8% |
| 88 | 8% | 92% | 8% |
| 89 | 8% | 92% | 8% |
| 90 | 8% | 92% | 8% |
| 91 | 8% | 92% | 8% |
| 92 | 8% | 92% | 8% |
| 93 | 8% | 92% | 8% |
| 94 | 8% | 92% | 8% |
| 95 | 8% | 92% | 8% |
| 96 | 8% | 92% | 8% |
| 97 | 8% | 92% | 8% |
| 98 | 8% | 92% | 8% |
| 99 | 8% | 92% | 8% |
| 100 | 8% | 92% | 8% |

Supplementary Table 9a The results of commercial CPMs in *mat*K 923

| sample | Three parallels (A/G, %) | | | Average value (%) | Actual value of *F. hupehensis* (g) | Total amounts (g) |
| --- | --- | --- | --- | --- | --- | --- |
|  | 1 | 2 | 3 |  |  |  |
| SDCB-1 | 50/50 | 52/48 | 52/48 | 51/49 | 0.3001 | 0.5846 |
| SDCB-2 | 50/50 | 51/49 | 51/49 | 51/49 | 0.3001 | 0.5923 |
| SDCB-3 | 50/50 | 50/50 | 52/48 | 51/49 | 0.3001 | 0.5923 |
| SDCB-4 | 51/49 | 51/49 | 52/48 | 51/49 | 0.3001 | 0.5846 |
| SDCB-5 | 52/48 | 52/48 | 51/49 | 52/48 | 0.3002 | 0.5810 |
| SDCB-6 | 51/49 | 50/50 | 50/50 | 50/50 | 0.3002 | 0.5964 |
| SDCB-7 | 51/49 | 50/50 | 51/49 | 51/49 | 0.3001 | 0.5923 |
| SDCB-8 | 52/48 | 52/48 | 53/47 | 52/48 | 0.3002 | 0.5736 |

Supplementary Table 9b The results of commercial CPMs in ITS 341

| sample | Three parallels (T/C, %) | | | Average value (%) | Amounts of FCB (g) |
| --- | --- | --- | --- | --- | --- |
|  | 1 | 2 | 3 |  |  |
| SDCB-1 | 40/60 | 39/61 | 40/60 | 40/60 | 0.2319 |
| SDCB-2 | 38/62 | 38/62 | 39/61 | 38/62 | 0.2270 |
| SDCB-3 | 50/50 | 49/51 | 49/61 | 49/51 | 0.2922 |
| SDCB-4 | 48/52 | 50/50 | 50/50 | 49/51 | 0.2884 |
| SDCB-5 | 49/51 | 48/52 | 48/52 | 48/52 | 0.2808 |
| SDCB-6 | 35/65 | 34/66 | 34/66 | 34/66 | 0.1969 |
| SDCB-7 | 34/66 | 35/65 | 33/67 | 34/66 | 0.2014 |
| SDCB-8 | 49/51 | 51/49 | 49/51 | 50/50 | 0.2849 |

Supplementary Table 9c The results of commercial CPMs in ITS 366

| sample | Three parallels (G/A, %) | | | Average value (%) | Amounts of adulterant (g) |
| --- | --- | --- | --- | --- | --- |
|  | 1 | 2 | 3 |  |  |
| SDCB-1 | 9/91 | 9/91 | 10/90 | 9/91 | 0.0546 |
| SDCB-2 | 11/89 | 11/89 | 12/88 | 11/89 | 0.0671 |
| SDCB-3 | 1/99 | 0/100 | 1/99 | 1/99 | / |
| SDCB-4 | 1/99 | 0/100 | 2/98 | 1/99 | / |
| SDCB-5 | 2/98 | 1/99 | 2/98 | 2/98 | / |
| SDCB-6 | 15/85 | 16/84 | 15/85 | 15/85 | 0.0915 |
| SDCB-7 | 14/86 | 16/84 | 14/86 | 15/85 | 0.0869 |
| SDCB-8 | 2/98 | 1/99 | 1/100 | 1/99 | / |
